# Protein Kinase C-Delta Mediates Cell Cycle Reentry and Apoptosis Induced by Amyloid-Beta Peptide in Post-Mitotic Cortical Neurons

**DOI:** 10.3390/ijms25179626

**Published:** 2024-09-05

**Authors:** Ming-Hsuan Wu, A-Ching Chao, Yi-Heng Hsieh, You Lien, Yi-Chun Lin, Ding-I Yang

**Affiliations:** 1 Institute of Brain Science, National Yang Ming Chiao Tung University, Taipei 112304, Taiwan; mission54321zack@gmail.com (M.-H.W.); p400540226@gmail.com (Y.-H.H.); as980938@gmail.com (Y.L.); 2 Department of Neurology, Kaohsiung Medical University Hospital, Kaohsiung 807377, Taiwan; achch@cc.kmu.edu.tw; 3 Department of Neurology, College of Medicine, Kaohsiung Medical University, Kaohsiung 807378, Taiwan; 4 Department of Sports Medicine, College of Medicine, Kaohsiung Medical University, Kaohsiung 807378, Taiwan; 5 Department of Neurology, Taipei City Hospital Renai Branch, Taipei 106243, Taiwan; 6 Brain Research Center, National Yang Ming Chiao Tung University, Taipei 112304, Taiwan

**Keywords:** Alzheimer’s disease, calpain, caspase-3, cyclin-dependent kinase-5 (CDK5), p53-upregulated modulator of apoptosis (PUMA), signal transducers and activators of transcription-3 (STAT3)

## Abstract

Amyloid-beta peptide (Aβ) is a neurotoxic constituent of senile plaques in the brains of Alzheimer’s disease (AD) patients. The detailed mechanisms by which protein kinase C-delta (PKCδ) contributes to Aβ toxicity is not yet entirely understood. Using fully differentiated primary rat cortical neurons, we found that inhibition of Aβ25-35-induced PKCδ increased cell viability with restoration of neuronal morphology. Using cyclin D1, proliferating cell nuclear antigen (PCNA), and histone H3 phosphorylated at Ser-10 (p-Histone H3) as the respective markers for the G1-, S-, and G2/M-phases, PKCδ inhibition mitigated cell cycle reentry (CCR) and subsequent caspase-3 cleavage induced by both Aβ25-35 and Aβ1-42 in the post-mitotic cortical neurons. Upstream of PKCδ, signal transducers and activators of transcription (STAT)-3 mediated PKCδ induction, CCR, and caspase-3 cleavage upon Aβ exposure. Downstream of PKCδ, aberrant neuronal CCR was triggered by overactivating cyclin-dependent kinase-5 (CDK5) via calpain2-dependent p35 cleavage into p25. Finally, PKCδ and CDK5 also contributed to Aβ25-35 induction of p53-upregulated modulator of apoptosis (PUMA) in cortical neurons. Together, we demonstrated that, in the post-mitotic neurons exposed to Aβs, STAT3-dependent PKCδ expression triggers calpain2-mediated p35 cleavage into p25 to overactivate CDK5, thus leading to aberrant CCR, PUMA induction, caspase-3 cleavage, and ultimately apoptosis.

## 1. Introduction

Alzheimer’s disease (AD) is the most common neurodegenerative disorder in the elderly. Although the detailed pathogenic mechanisms of AD are still not fully understood, extracellular senile plaques containing aggregated amyloid-beta peptide (Aβ) derived from amyloid precursor protein (APP) are widely acknowledged as a pathological hallmark in AD brains [1]. Aberrant cell cycle reentry (CCR) consequently with induction of apoptosis in post-mitotic neurons, which remain quiescent at the G0 phase once completely differentiated, is a neurotoxic mechanism of Aβs [2]. Several studies suggest the correlation between CCR and neuronal loss in AD. For example, post-mitotic AD neurons express marker proteins involved in cell cycle progression [3,4]. CCR and accumulation of cyclin D1, one of the proteins required for G1/S transition, resulting in neuronal death have been reported in the brains of AD patients carrying presenilin-1 gene mutations [5]. AD neurons undergoing CCR are usually arrested at the G2/M transition checkpoint [6]. Despite these observations, detailed molecular mechanisms underlying Aβ-triggered CCR in differentiated neurons remain to be fully delineated.

Protein kinase C (PKC) isozymes belong to a phospholipid-dependent family of serine/threonine protein kinases that plays an important role in the regulation of cellular proliferation and differentiation [7]. Twelve PKC isozymes, which are categorized into classical, novel, and atypical PKCs, play an important role in various neurodegenerative diseases, including AD [8]. Among these PKC isoforms, one of the novel PKCs, or PKCδ, may play a detrimental role because curcumin-induced PKCδ degradation has been reported to enhance spatial learning capability in aged rats and adults [9]. In the brains of AD patients, expression of PKCδ is increased compared with the non-AD controls; PKCδ inhibition can also reduce Aβ production and rescue cognitive deficits in APPswe/PS1dE9 transgenic mice [10]. These studies together suggest that PKCδ may have a negative impact in brain aging and neurodegenerative disorders including AD.

Cyclin-dependent kinases (CDKs) are proline-directed serine/threonine kinases that are activated by binding with regulatory proteins. Highly conserved throughout evolution, CDKs are present in species from *Saccharomyces cerevisiae* to humans [11]. Most CDKs are activated through association and phosphorylation of their T-loop by a cyclin; however, CDK5 is atypical because it is activated by neither cyclin association nor phosphorylation, despite its high homology in amino acid sequences with other CDKs [12]; instead, CDK5 is activated through binding with the regulatory proteins p35 or p39 specifically expressed in neuronal cells [13,14]. Increases in calcium concentration lead to calpain-dependent cleavage of p35 and p39 into their respective cleaved counterparts p25 and p29, thereby hyperactivating CDK5 with sustained enzymatic activities [15]. In the nervous system, CDK5 plays an important role in neuronal survival, neural development, mitochondrial fission, phosphorylation of cytoskeletal proteins, and synaptic plasticity [16,17]. Notably, Aβs may overactivate CDK5, which eventually results in aberrant phosphorylation of AD-related protein substrates like tau and APP [18,19,20]. Another pathological substrate of CDK5 is p53 [21], whose downstream target, p53-upregulated modulator of apoptosis (PUMA), also plays an essential role in the caspase-3-dependent apoptosis induced by Aβs in vitro [22].

Signal transducer and activator of transcription 3 (STAT3) is a transcriptional activator recently implicated in AD, with contradictory findings. First, STAT3 may contribute to AD pathogenesis via reactive astrogliosis with neuroinflammation or affecting APP processing. For example, activated STAT3 contributes to checkpoint kinase 1 (ChK1)-mediated reactive astrogliosis, neuronal degeneration, and exacerbation of AD [23]. Intrahippocampal microinjection of oligomeric Aβ into rat brains leads to STAT3 induction along with reactive astrogliosis and neuronal death [24]. Reactive astrocytes in the AD brain are induced via STAT3, and the impairments in learning and memory observed in 5 × FAD mice can be rescued by STAT3 inhibition [25]. Notably, a STAT3-specific inhibitor may recover cognitive functions and augment cerebral blood flow in the 5 × E4 mice, along with improvements in oxidative stress and neuroinflammation [26]. Finally, several neuroprotective agents appear to exert their beneficial effects in AD models by blocking the STAT3 signaling pathway to suppress neuroinflammatory responses [27] or affecting APP processing with decreased Aβ production [28]. These reports seem to support a detrimental role of STAT3 activation in AD. Contradictorily, however, STAT3 overexpression restores synaptic loss and ameliorates cognitive deficits through modulation of the N-methyl-D-aspartate receptors (NMDARs) in an AD animal model [29], suggesting a beneficial role of STAT3 in AD. Despite these aforementioned studies, whether STAT3 directly contributes to neurotoxicity of existing Aβs and the potential underlying mechanisms are less well understood.

Despite correlative clinical evidence linking PKCδ to AD and the observations that PKCδ inhibition can attenuate Aβ production in animal models [10], whether PKCδ directly contributes to Aβ neurotoxicity remains to be determined. In the present study, we therefore asked whether Aβ induces expression of PKCδ and investigated its upstream mediator, namely STAT3, in primary cultures of rat cortical neurons. Extended from our previous report that CDK5 contributes to Aβ-induced CCR and caspase-dependent apoptosis in post-mitotic neurons [30], we also investigated whether the downstream mediators like CDK5, its neuronal regulatory proteins p35/p25, calpains, and PUMA are involved in PKCδ-dependent neurotoxicity.

## 2. Results

### 2.1. PKCδ Inhibition Exerts Neuroprotective Effects against Aβ25-35 Toxicity

**Figure 1:**
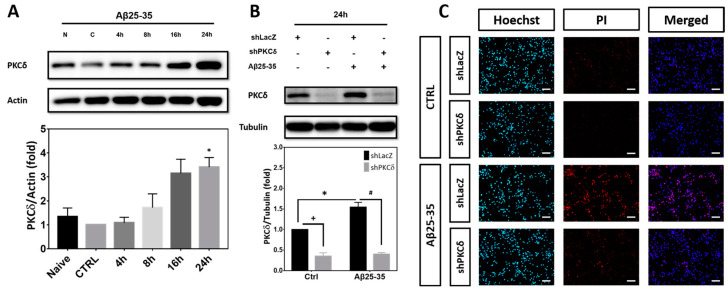

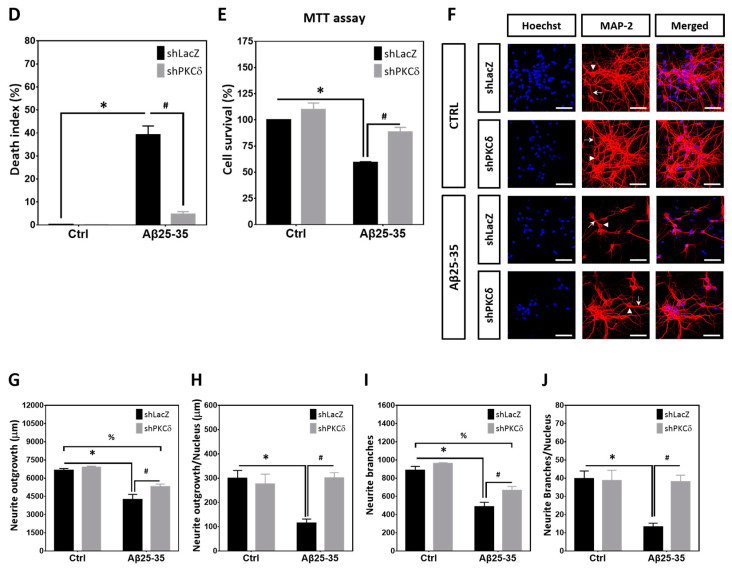
PKCδ inhibition exerts protective effects against neurotoxicity induced by Aβ25-35. (**A**) Primary cortical cultures were treated with 10 μM Aβ25-35 for indicated times before detection of PKCδ by Western blotting. β-Actin served as the internal control for equal loading of proteins in each lane. Mean ± S.E.M. from N = 4. * denotes *p* < 0.05 compared with the corresponding Naïve (no treatment) and control (CTRL, treated with the same volume of vehicle ddH_2_O) cultures without Aβ25-35 treatment. (**B**) Primary cortical neurons were transfected with LacZ-shRNA or PKCδ-shRNA for 24 h, followed by exposure to 10 μM Aβ25-35 for an additional 24 h (**B**,**F**–**J**) or 48 h (**C**–**E**). This was followed by detection of PKCδ by Western blotting (B), determination of cell viability by Hoechst/PI double staining (**C**,**D**) or MTT assays (**E**), and examination of neuronal morphology (**F**–**J**). For neuronal morphology, cortical cultures were immunostained with an antibody against MAP-2 (red) to label the mature neurons; Hoechst 33258 (blue) served as the counterstaining. The arrowheads and arrows denote the representative neuronal soma and their neurites, respectively. Scale bar in (C) and (F) = 50 μm. Representative micrographs from 3 independent experiments with similar results are shown in (**F**). Quantitative analyses of the neurite outgrowth and neurite branch numbers are shown in (**G**–**J**). Mean ± S.E.M. from N = 4 in (**B**), N = 3 in (**C**, **D**), N = 4 in (**E**), and N = 3 in (**G**-**J**). *, #, and + all denote *p* < 0.05.

Heightened expression of PKCδ has previously been reported in AD [10]. We also observed that Aβ25-35 (10 μM) time-dependently increased PKCδ expression in primary rat cortical neurons, reaching the maximal levels at 16–24 h (Figure 1A). To investigate the neuroprotective effects of PKCδ inhibition, cortical cultures were transfected with lentivirus expressing the shRNA targeting PKCδ, followed by exposure to Aβ25-35. Results indicated that expression of PKCδ was significantly increased in the primary cortical cultures treated with Aβ25-35 for 24 h; further, expression of both the endogenous as well as the Aβ25-35-induced PKCδ was markedly downregulated by its shRNA (Figure 1B). Consistent with a neuroprotective effect, both propidium iodide (PI)/Hoechst double staining (Figure 1C,D) and MTT (3-(4,5-dimethylthiazol-2-yl)-2,5-diphenyltetrazolium bromide) reduction assays (Figure 1E) showed that PKCδ inhibition increased cell viability of cortical cultures exposed to Aβ25-35 for 48 h. Immunostaining with the antibody against microtubule-associated protein-2 (MAP-2), which is a protein marker for neuronal dendrites, further revealed that PKCδ inhibition restored Aβ25-35-induced morphological damages in differentiated neurons (Figure 1F); quantitative analyses confirmed that PKCδ inhibition restored total neurite outgrowth (Figure 1G), neurite outgrowth per neuron (Figure 1H), total numbers of neurite branches (Figure 1I), and the numbers of neurite branches per neuron (Figure 1J). Taken together, these results indicate that Aβ25-35 enhances PKCδ expression, and its inhibition by shRNA mitigates Aβ25-35 toxicity, with restoration of neuronal morphology in vitro.

### 2.2. PKCδ Inhibition Blocks Aberrant CCR and Apoptosis Induced by Aβ25–35 in Differentiated Cortical Neurons

**Figure 2:**
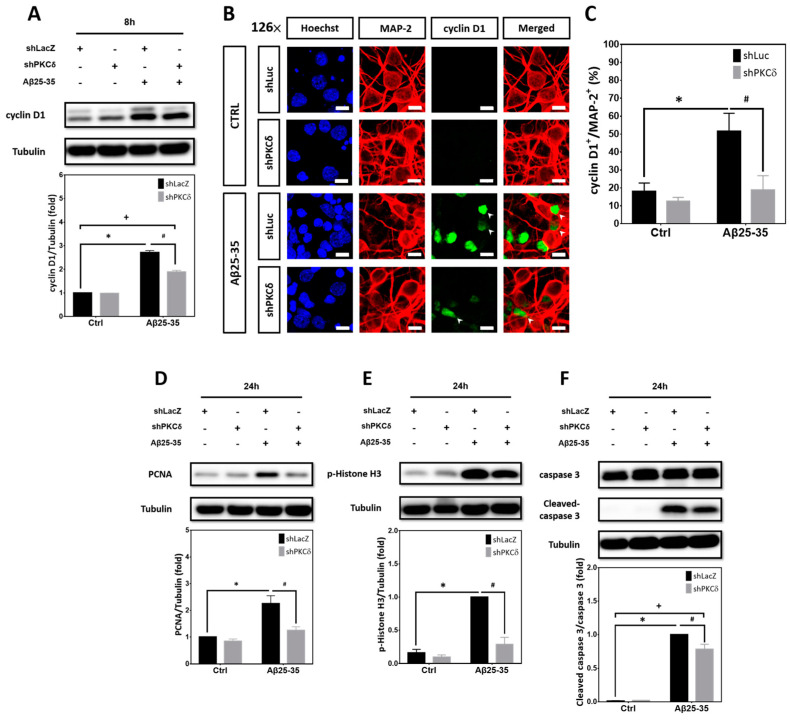

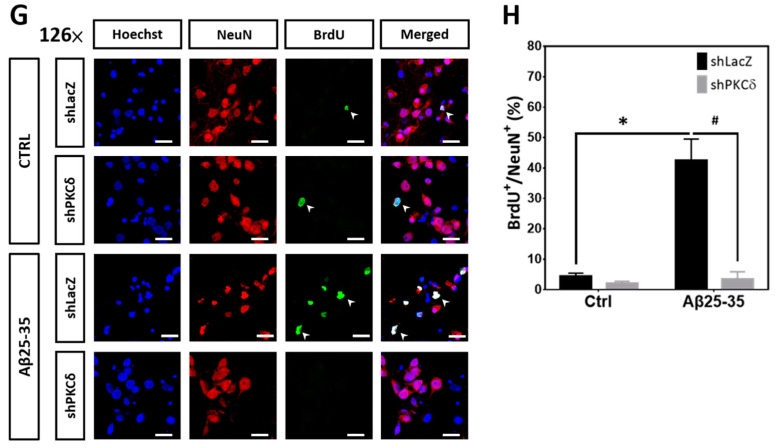
PKCδ inhibition attenuates Aβ25-35-mediated CCR and caspase-3 cleavage in post-mitotic neurons. (**A**–**C**) Primary cortical cultures were transfected with the control LacZ-shRNA or Luc-shRNA, as indicated in the figure panels above, or PKCδ-shRNA for 24 h, followed by exposure to 10 μM Aβ25-35 for an additional 8 h, before detection of cyclin D1 by Western blotting in (**A**) or immunostaining with the antibodies against cyclin D1 (green) and MAP-2 (red) in (**B**,**C**); Hoechst 33258 (blue) served as counterstaining. The arrowheads denote the representative cyclin D1^+^ neurons. Scale bars = 10 μm. Representative micrographs from 3 independent experiments with similar results are shown in (**B**); quantitative analyses of the cyclin D1^+^/MAP-2^+^ cells are shown in (**C**). Mean ± S.E.M. from N = 3 in (**A**) and (**C**). *, #, and + all denote *p* < 0.05. (**D**–**F**) The experimental conditions were the same as described in (**A**–**C**) except cells were treated with Aβ25-35 for an additional 24 h before detection of PCNA (**D**), p-Histone H3 (**E**), as well as pro- and cleaved caspase 3 (**F**) by Western blotting. α-Tubulin served as the internal control for equal loading of proteins in each lane. Mean ± S.E.M. from N = 5 in (**D**) and N = 3 in (**E**,**F**). *, #, and + all denote *p* < 0.05. (**G**,**H**) The experimental conditions were the same as described in (**D**-**F**) except that cells were subjected to immunostaining with the antibodies against BrdU (green) and NeuN (red); Hoechst 33258 (blue) served as counterstaining. The arrowheads denote the representative BrdU^+^ neurons. Scale bars = 20 μm. Representative micrographs from 3 independent experiments with similar results are shown in (**G**); quantitative analyses of the BrdU^+^/NeuN^+^ cells are shown in (**H**). Mean ± S.E.M. from N = 3 in (**H**). * and # denote *p* < 0.05.

For entry of quiescent cells at G0 into the G1 state, CDK4/6 is associated with D-type cyclin, including cyclin-D1; the cyclin-D/CDK4/6 complex is required for the phosphorylation of retinoblastoma (Rb) protein, which causes the release of transcription factor E2F to permit cell cycle progression through G1/S transition. Proliferating cell nuclear antigen (PCNA) is an auxiliary protein of DNA polymerase delta, important for DNA replication during the S phase. Histone H3 phosphorylated at Ser-10 (p-Histone H3) is crucial for dynamic chromatin condensation during the mitotic phase [31]. To investigate the underlying neuroprotective mechanisms, cyclin D1, PCNA, and p-Histone H3 were selected as the respective markers for the G1, S, and M phases during cell cycle progression, as reported in our previous studies [30,32], to examine whether PKCδ inhibition suppresses Aβ25-35-induced neuronal CCR and apoptosis. We found that Aβ25-35 significantly increased expression of cyclin D1 that was suppressed by PKCδ knockdown (Figure 2A); similar results were observed when the cortical cultures were subjected to immunocytochemical staining to colocalize cyclin D1 in the MAP-2^+^ cells, indicative of cell cycle reactivation in the post-mitotic cortical neurons (Figure 2B,C). Furthermore, Aβ25-35 also enhanced expression of other cell cycle markers such as PCNA (Figure 2D) and p-Histone H3 (Figure 2E) in the differentiated cortical cultures based on Western blotting; both were suppressed by PKCδ knockdown. Consistent with a neurotoxic effect of neuronal CCR, Aβ25-35 enhanced caspase-3 cleavage, as an index for apoptosis, which was also significantly attenuated by PKCδ inhibition (Figure 2F). Next, to reveal de novo DNA synthesis during the S phase of cell cycle reactivation in the post-mitotic neurons, BrdU (5-bromo-2′-deoxyuridine) incorporation coupled with immunocytochemistry using the neuronal marker NeuN was performed. Representative micrographs shown in Figure 2G clearly indicate that Aβ25-35 increased the numbers of BrdU^+^/NeuN^+^ cells, implying enhanced de novo DNA synthesis in the differentiated neurons, which were completely abolished by PKCδ inhibition (Figure 2H). These results together demonstrate that PKCδ downregulation blocks Aβ25-35-induced neuronal CCR and caspase-dependent apoptosis in fully differentiated cortical neurons.

### 2.3. Pharmacological Activation of PKCs by PMA Is Sufficient to Upregulate Expression of Cell Cycle and Apoptotic Markers That Can Be Blocked by PKCδ Knockdown

**Figure 3:**
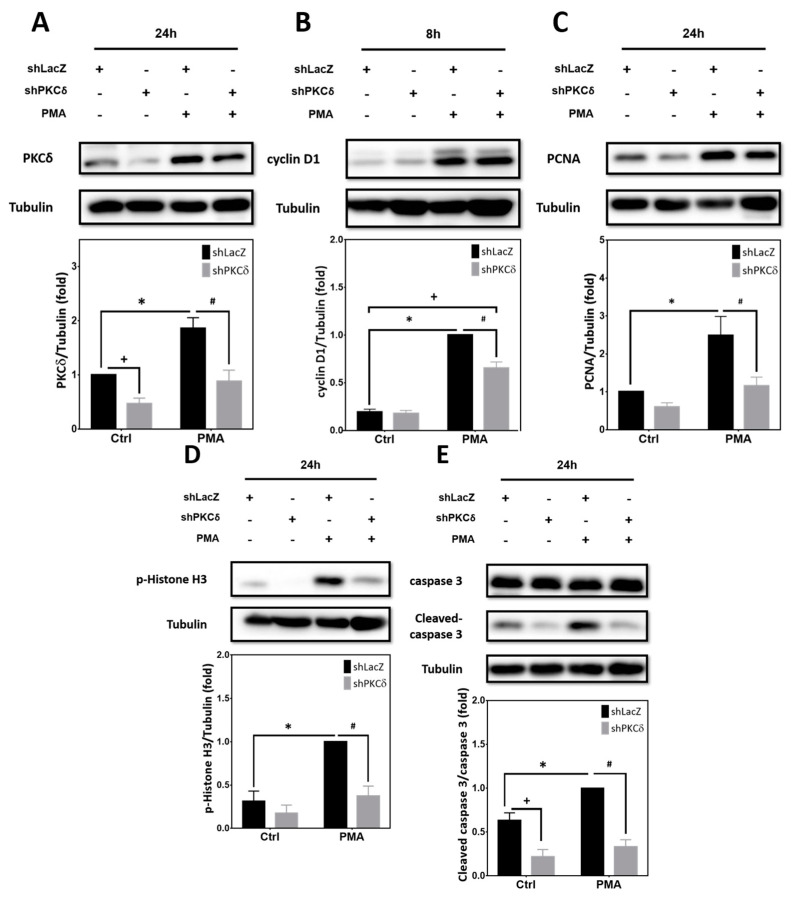
PKCδ activation by PMA alone is sufficient to trigger CCR and caspase-3 cleavage in post-mitotic neurons. Primary cortical neurons were transfected with LacZ-shRNA or PKCδ-shRNA for 24 h, followed by exposure to 40 ng/mL PMA for an additional 8 h, before detection of cyclin D1 (**B**) or an additional 24 h before detection of PKCδ (**A**), PCNA (**C**), and p-Histone H3 (**D**), as well as pro- and cleaved caspase 3 (**E**). α-Tubulin served as the internal control for equal loading of proteins in each lane. Mean ± S.E.M. from N = 3. *, #, and + all denote *p* < 0.05.

Although Aβ25-35-mediated neuronal CCR and apoptosis are mediated by PKCδ, whether activation of PKCδ alone is sufficient to trigger these effects independent of Aβ in post-mitotic neurons remains unclear. To address this issue, cortical cultures were transfected with lentivirus expressing the shRNA targeting PKCδ prior to treatment with phorbol-12-myristate-13-acetate (PMA), a phorbol ester capable of PKC activation [33]. As shown in Figure 3A, expression of PKCδ was significantly increased by PMA treatment for 24 h, and was downregulated by PKCδ shRNA. Furthermore, PMA markedly enhanced the expression of cyclin D1 (Figure 3B), PCNA (Figure 3C), p-Histone H3 (Figure 3D), and caspase-3 cleavage (Figure 3E), all of which were suppressed by PKCδ knockdown. Collectively, results shown in Figure 2 and Figure 3 reveal that PKCδ induction by Aβ25-35 is necessary for aberrant CCR and apoptosis in post-mitotic neurons; furthermore, PMA-mediated PKCδ activation alone is sufficient to directly trigger these neuronal effects, even without Aβ25-35.

### 2.4. PKCδ Inhibition Lessens Aβ25-35- and PMA-Mediated CCR by Attenuating CDK5 Hyperactivation in Primary Cortical Cultures

**Figure 4:**
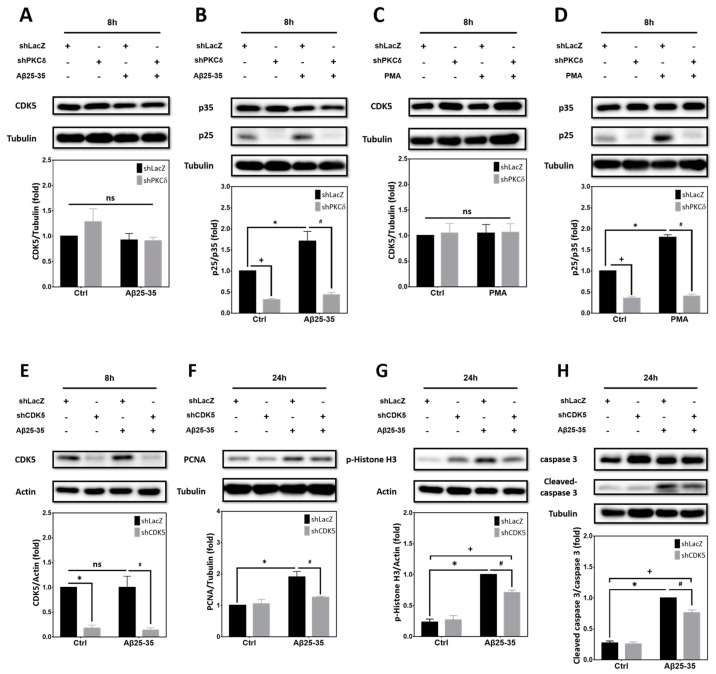
Aβ25-35/PMA induce PKCδ-dependent p35 cleavage into p25 and Aβ25-35 induces CDK5-dependent neuronal CCR with caspase-3 cleavage. (**A**–**D**) Primary cortical neurons were transfected with LacZ-shRNA or PKCδ-shRNA for 24 h, followed by exposure to 10 μM Aβ25-35 (**A**,**B**) or 40 ng/mL PMA (**C**,**D**) for an additional 8 h, before detection of CDK5 (**A**,**C**) or p35/p25 (**B**,**D**). (**E**–**H**) Cultures were transfected with LacZ-shRNA or CDK5-shRNA for 24 h, followed by exposure to 10 μM Aβ25-35 for an additional 8 h, before detection of CDK5 (**E**) or 24 h before detection of PCNA (**F**), and p-Histone H3 (**G**), as well as pro- and cleaved caspase-3 (**H**). β-Actin and α-tubulin served as the internal control for equal loading of proteins in each lane. Mean ± S.E.M. from N = 3 in (**A**), N = 5 in (**B**), N = 3 in (**C**,**D**), N = 4 in (**E**), and N = 3 in (**F**–**H**). *, #, and + all denote *p* < 0.05; “ns” denotes “not significant”.

We have previously shown that CDK5 participates in Aβ-induced aberrant CCR and neuronal apoptosis [30]. However, whether PKCδ is involved in this mechanism remains unclear. We therefore tested the effects of knocking down PKCδ on CDK5 activation and neuronal CCR. Results indicated that expression of CDK5 was not significantly altered by Aβ25-35 or PKCδ shRNA (Figure 4A). However, Aβ25-35-induced cleavage of p35 into p25, an indicator for CDK5 overactivation, was markedly downregulated by PKCδ shRNA (Figure 4B). Similarly, neither PMA nor PKCδ shRNA affected CDK5 expression (Figure 4C), yet PMA was sufficient to trigger p35 cleavage into p25, which was also completely blocked by PKCδ knockdown. Indeed, basal levels of p25 were substantially repressed by PKCδ shRNA in the absence of Aβ25-35 (Figure 4B) or PMA (Figure 4D). To further clarify the role of CDK5, shRNA was applied to achieve gene-specific knockdown in cortical cultures. Results indicated that the CDK5 shRNA effectively suppressed its protein expression, irrespective of Aβ25-35 (Figure 4E). More importantly, CDK5 knockdown attenuated Aβ25-35-dependent induction of PCNA (Figure 4F), p-Histone H3 (Figure 4G), and caspase-3 cleavage (Figure 4H). Taken together, results shown in Figure 2, Figure 3 and Figure 4 support the signaling cascade of “Aβ → PKCδ → CDK5/p25 → CCR → caspase-3 cleavage → apoptosis” in the differentiated post-mitotic cortical neurons.

### 2.5. PKCδ Inhibition Blocks Neuronal CCR and Apoptosis Induced by Aβ1–42

**Figure 5:**
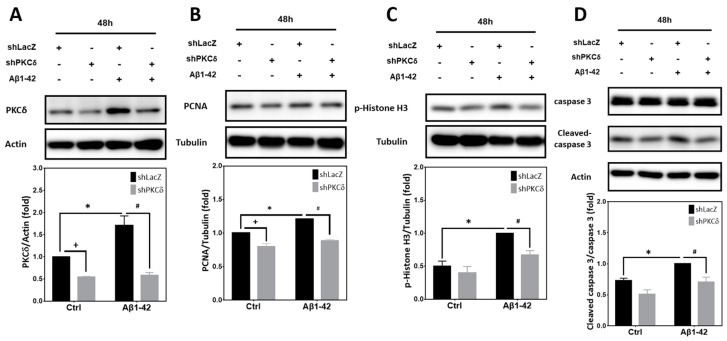
PKCδ inhibition attenuates Aβ1-42-mediated CCR and caspase-3 cleavage in post-mitotic neurons. Primary cortical neurons were transfected with LacZ-shRNA or PKCδ-shRNA for 24 h, followed by exposure to 5 μM Aβ1-42 for an additional 48 h, before detection of PKCδ (**A**), PCNA (**B**), and p-Histone H3 (**C**), as well as pro- and cleaved caspase-3 (**D**). β-Actin and α-tubulin served as the internal control for equal loading of proteins. Mean ± S.E.M. from N = 3 in (**A**–**C**) and N = 4 in (**D**). *, #, and + all denote *p* < 0.05.

In addition to Aβ25-35, we also examined whether PKCδ inhibition suppresses neuronal CCR and apoptosis induced by Aβ1-42, the pathologically more relevant Aβ species. Similar to the results derived from Aβ25-35 exposure, Aβ1-42 significantly enhanced expression of PKCδ (Figure 5A), PCNA (Figure 5B), p-Histone H3 (Figure 5C), and caspase-3 cleavage (Figure 5D), all of which were attenuated by PKCδ knockdown. These results confirm that the observed PKCδ effects on neuronal CCR and apoptosis can also be extended to Aβ1-42.

### 2.6. STAT3 Contributes to Aβ25-35- and PKCδ-Dependent Neuronal CCR and Apoptosis

**Figure 6:**
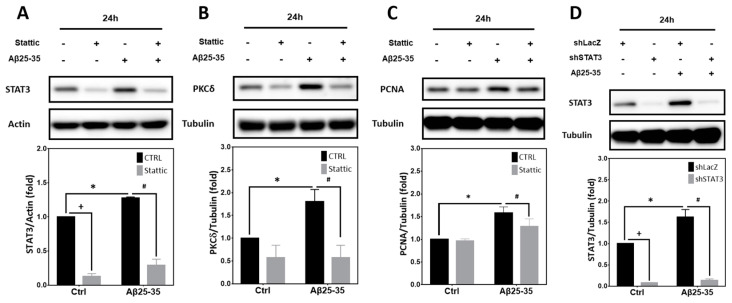

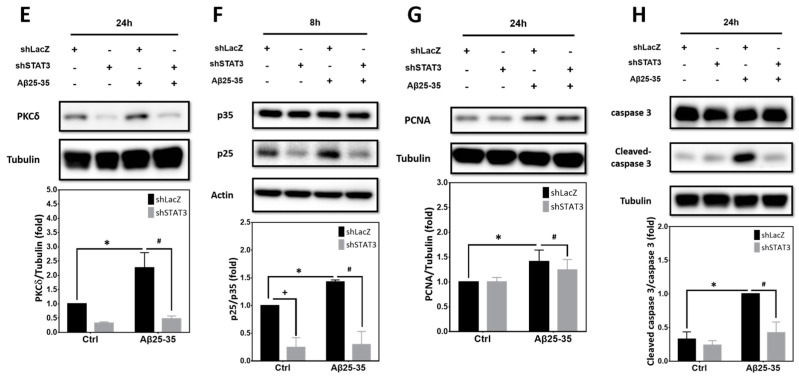
STAT3 inhibition attenuates Aβ25-35-mediated PKCδ induction, p35 cleavage into p25, neuronal CCR, and caspase-3 cleavage in the post-mitotic neurons. (**A**–**C**) Primary cortical neurons were exposed to 10 μM Aβ25-35 with or without 5 μM stattic for 24 h before detection of STAT3 (**A**), PKCδ (**B**), or PCNA (**C**). (**D**–**H**) Primary cortical neurons were transfected with LacZ-shRNA or STAT3-shRNA for 24 h, followed by exposure to 10 μM Aβ25-35 for an additional 8 h, before detection of p35/p25 (**F**) or 24 h before detection of STAT3 (**D**), PKCδ (**E**), and PCNA (**G**), as well as pro- and cleaved caspase 3 (**H**). β-Actin and α-tubulin served as the internal control for equal loading of proteins in each lane. Mean ± S.E.M. from N = 3 in (**A**), N = 4 in (**B**,**C**), N = 3 in (**D**), N = 4 in (**E**), and N = 3 in (**F**–**H**). *, #, and + all denote *p* < 0.05.

Previously it has been reported that phosphorylation of STAT3 by PKCδ leads to a negative regulation of STAT3 DNA binding and transcriptional activity [34]. However, whether STAT3 is involved in Aβ25-35- or PKCδ-dependent CCR in post-mitotic neurons remains unclear. As shown in Figure 6A, expression of the basal STAT3 proteins and those induced by Aβ25-35 was downregulated by stattic, a small-molecule inhibitor of STAT3 capable of suppressing its activation [25]. Further, stattic also inhibited Aβ25-35-mediated induction of PKCδ (Figure 6B) and the S-phase marker PCNA (Figure 6C). Similar to stattic, STAT3 shRNA, which effectively suppressed the basal as well as the Aβ25-35-induced STAT3 (Figure 6D), also attenuated Aβ25-35-dependent PKCδ induction (Figure 6E), p35 cleavage into p25 (Figure 6F), PCNA induction (Figure 6G), and caspase-3 cleavage (Figure 6H). That both pharmacological inhibition and gene-specific knockdown of STAT3 blocked PKCδ induction by Aβ25-35 strongly suggests that STAT3 acts upstream of PKCδ/CDK5/p25 to trigger neuronal CCR and caspase-3 cleavage.

### 2.7. Calpain2, but Not calpain1, Contributes to PKCδ-Dependent CCR Induced by Aβ25–35 in Primary Cortical Cultures

**Figure 7:**
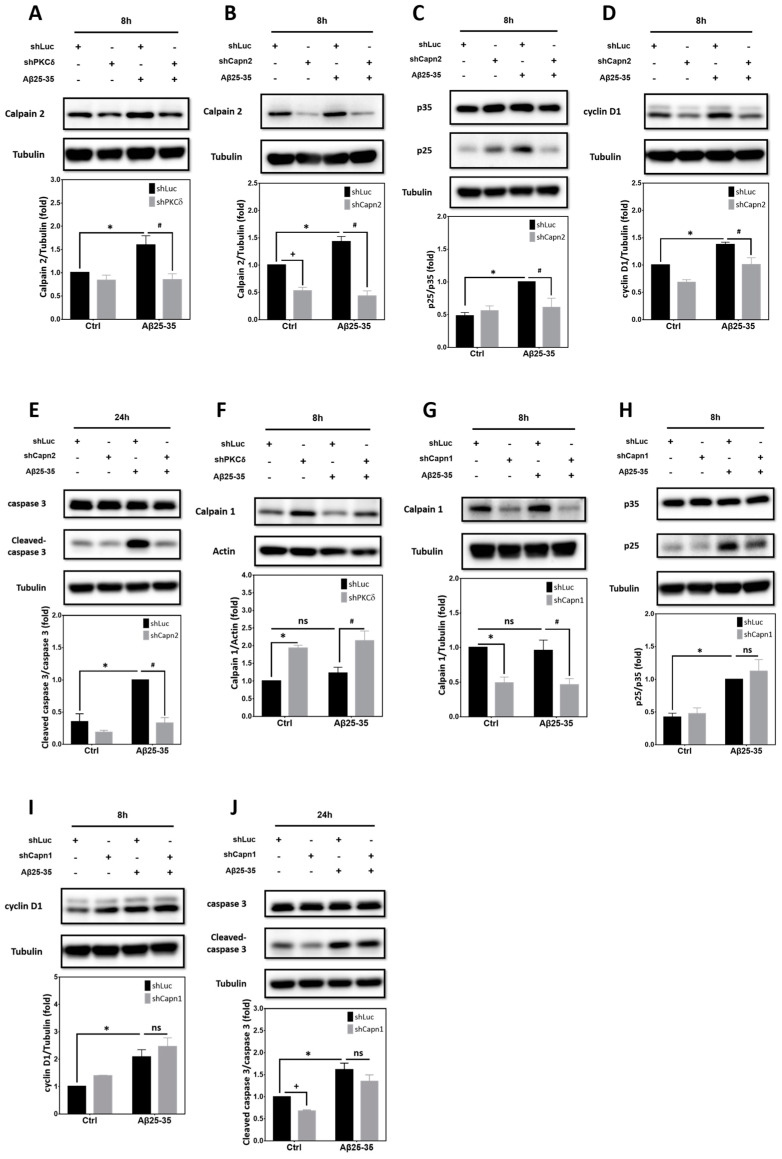
Aβ25-35-induced calpain2, but not calpain1, mediates PKCδ-dependent p35 cleavage into p25, neuronal CCR, and caspase-3 cleavage. (**A**) Primary cortical neurons were transfected with Luc-shRNA or PKCδ-shRNA for 24 h, followed by exposure to 10 μM Aβ25-35 for an additional 8 h, before detection of calpain2. (**B**-**E**) Primary cortical neurons were transfected with Luc-shRNA or calpain2-shRNA (shCapn2) for 24 h, followed by exposure to 10 μM Aβ25-35 for an additional 8 h (**B**–**D**) or 24 h (**E**), before detection of calpain2 (**B**), p35/p25 (**C**), and cyclin D1 (**D**), as well as pro- and cleaved caspase-3 (**E**). (**F**) Primary cortical neurons were transfected with Luc-shRNA or PKCδ-shRNA for 24 h, followed by exposure to 10 μM Aβ25-35 for an additional 8 h, before detection of calpain1. (**G**–**J**) Primary cortical neurons were transfected with Luc-shRNA or calpain1-shRNA (shCapn1) for 24 h, followed by exposure to 10 μM Aβ25-35 for an additional 8 h (**G**–**I**) or 24 h (**J**), before detection of calpain1 (**G**), p35/p25 (**H**), and cyclin D1 (**I**), as well as pro and cleaved caspase 3 (**J**). β-Actin and α-tubulin served as the internal control for equal loading of proteins in each lane. Mean ± S.E.M. from N = 5 in (**A**), N = 4 in (**B**, **C**), N = 3 in (**D**, **E**), N = 4 in (**F**), and N = 3 in (**G**-**J**). *, #, and + all denote *p* < 0.05; “ns” denotes “not significant”.

Calcium-activated neutral proteases, or calpains, are intracellular nonlysosomal cysteine proteases. Dysregulation of calcium homeostasis triggers pathological activation of calpain in several neurodegenerative diseases including AD [35]. Indeed, CDK5 is known to be hyperactivated by Aβ through calpain-mediated conversion of p35 to p25 in primary cortical neurons [36]. Two prototypical calpains exist that include μ-calpain (calpain1), activated by calcium at μM concentrations in vitro, and m-calpain (calpain2), requiring mM calcium for activation. We have previously reported that the calpain inhibitor III MDL28170, which inhibits both calpain1 and calpain2 as well as other proteases like cathepsin B, may block Aβ25-35-dependent p35 cleavage into p25 [30]. However, exactly which calpain(s) mediate Aβ25-35- and PKCδ-dependent CCR in the post-mitotic neurons remains unclear. We therefore took a gene-specific knockdown approach to identify the calpain involved in this pathway. Figure 7A reveals that induction of calpain2 by Aβ25-35 was downregulated by PKCδ shRNA; furthermore, calpain2 shRNA capable of suppressing both basal and the Aβ25-35-induced calpain2 expression (Figure 7B) also attenuated p35 cleavage into p25 (Figure 7C). Consistently, neuronal CCR and apoptosis, as, respectively, indicated by the induction of cyclin D1 (Figure 7D) and caspase-3 cleavage (Figure 7E), in the post-mitotic neurons were also alleviated by calpain2 inhibition. Unlike calpain2, however, expression of calpain1 was not significantly induced by Aβ25-35, nor was it inhibited by PKCδ shRNA (Figure 7F). Intriguingly, PKCδ knockdown indeed enhanced the expression of calpain1, irrespective of Aβ25-35 exposure (Figure 7F), implying that endogenous PKCδ may instead exert an inhibitory effect on calpain1 expression. Despite its effectiveness in suppressing both basal and Aβ25-35-induced calpain1 (Figure 7G), the calpain1 shRNA failed to significantly impact Aβ25-35-mediated p35 cleavage into p25 (Figure 7H) or the induction of cyclin D1 (Figure 7I), although calpain1 knockdown did slightly attenuate the endogenous, but not the Aβ25-35-induced, caspase-3 cleavage (Figure 7J). These results thus support the critical roles of calpain2 while excluding potential involvements of calpain1 in Aβ-induced neuronal CCR and apoptosis in post-mitotic cortical neurons.

### 2.8. Inhibition of PKCδ and CDK5 Downregulates PUMA Expression Induced by Aβ25–35

**Figure 8:**
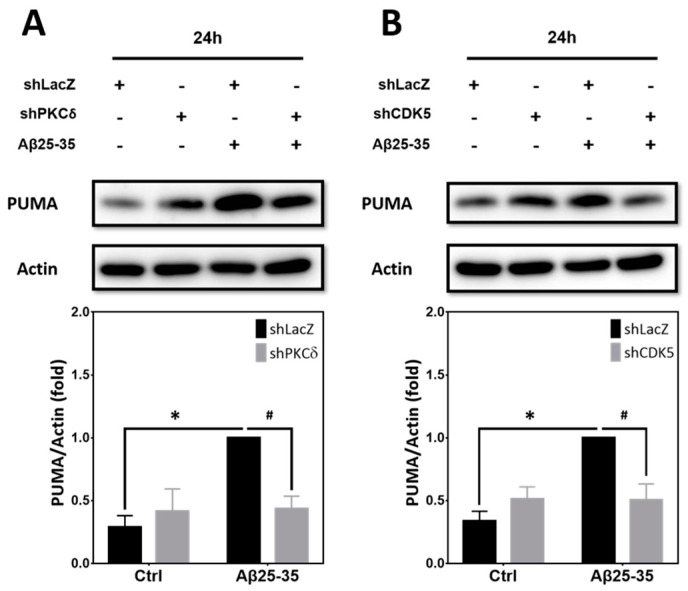
Aβ25-35 induces PKCδ- and CDK5-dependent expression of PUMA. (**A**,**B**) Primary cortical neurons were transfected with PKCδ-shRNA (**A**) or CDK5-shRNA (**B**) for 24 h, followed by exposure to 10 μM Aβ25-35 for an additional 24 h, before detection of PUMA by Western blotting. Cortical cultures transfected with LacZ-shRNA served as the negative control for lentivirus-mediated gene transfer. β-Actin served as the internal control for equal loading of proteins in each lane. Mean ± S.E.M. from N = 3. * and # denote *p* < 0.05.

Aβ25-35 and Aβ1-42 have been shown to trigger CDK5-dependent phosphorylation and stabilization of p53, leading to mitochondrial dysfunction and neuronal apoptosis in primary cultures of mouse cortical neurons [21]. An earlier study also reported that the p53-upregulated modulator of apoptosis (PUMA), one of the p53 downstream targets, plays an essential role in the caspase-3-dependent apoptosis induced by Aβs in primary hippocampal neurons [22]. We therefore tested whether PUMA also participates in PKCδ/CDK5-mediated neuronal CCR and apoptosis. In line with the prior research, PUMA was significantly increased in the primary cortical cultures exposed to Aβ25-35 for 24 h; notably, Aβ25-35-induced PUMA expression was attenuated by PKCδ shRNA (Figure 8A) as well as CDK5 shRNA (Figure 8B). 

## 3. Discussion

PKCδ has been implicated in AD pathogenesis. For example, increased PKCδ levels are observed in the brain of AD patients and may contribute to heightened expression of the beta-site APP cleaving enzyme-1 (BACE1), and, consistently, knockdown of PKCδ reduces Aβ production in APPswe/PS1dE9 transgenic mice [10]. Further, Aβ stimulation of microglia increases PKCδ expression and secretion, thus upregulating the nuclear factor kappa B (NF-κB) pathway, along with heightened production of inflammatory cytokines [37]. PKCδ expression was also enhanced in the 3 × Tg AD mice following laparotomy, and its pharmacological inhibition significantly increased expression of synaptic proteins in the synaptosome fractions derived from the hippocampal CA3 region of mouse brains [38]. In our study, we also observed elevated PKCδ levels upon exposure to Aβ25-35 (Figure 1A) and Aβ1-42 (Figure 5A) in primary cortical neurons. In addition to modulating production of Aβ and secretion of proinflammatory cytokines, we also report a novel mechanism of PKCδ in relation to AD pathogenesis, wherein it directly mediates the neurotoxic effects of the existing Aβs by triggering neuronal CCR, followed by apoptosis. Apart from negatively impacting on neurons and microglia, Aβ1–40 induces platelet adhesion, which is a vascular pathology observed in AD via a PKCδ-dependent signaling pathway [39]. These previous works and our current findings together suggest a detrimental effect of elevated PKCδ in AD, albeit via different mechanisms. Given this contention, it is predicted that downregulation of PKCδ expression or pharmacological inhibition of its biological function by small-molecule inhibitors should exert beneficial effects in AD. Rottlerin is a compound from *Mallotus philippinensis* that can inhibit PKCδ, with an IC50 of 3-6 μM [40]. In the AD transgenic mouse line of APPswe/PS1dE9, rottlerin-mediated PKCδ inhibition rescued cognitive deficits while reducing Aβ generation and deposition [10], suggesting that PKCδ may be a potential therapeutic target for AD. Another small-molecule compound, CGX1037, has been proposed to be a selective inhibitor for PKCδ in platelets [41]. However, its potential application in nervous systems still remains unclear.

**Figure 9:**
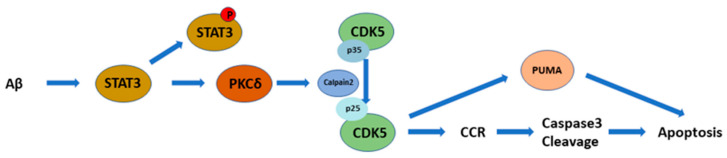
A diagram showing the proposed signal transduction pathway. In the fully differentiated post-mitotic neurons, Aβs trigger STAT3-dependent PKCδ induction to enhance calpain2-mediated cleavage of p35 into p25, with resultant CDK5 hyperactivation, thereby inducing aberrant CCR and expression of the pro-apoptotic PUMA as well as the cleaved caspase-3 to ultimately culminate in neuronal apoptosis.

In the present study, we presented experimental evidence supporting the signaling cascade of “Aβ → STAT3 → PKCδ → CDK5/p25 → CCR/PUMA/caspase-3 → apoptosis” in post-mitotic primary cortical neurons (Figure 9). It should be noted, however, that these conclusions mainly rely on the change in protein levels shown in Western blotting experiments. In our earlier reports, we applied several different techniques to firmly establish the notion of Aβ25-35/1-42-induced neuronal CCR [30,32,42]. These include Western blotting to detect the expression of protein markers specific for each cell cycle phase (such as cyclin D1, the retinoblastoma protein or pRb phosphorylated at Ser-807/811, PCNA, and p-Histone H3), immunocytochemical colocalization of these cell cycle markers in MAP-2^+^ or NeuN^+^ mature neurons, BrdU incorporation to detect de novo DNA synthesis in NeuN^+^ neurons, as well as flow cytometry to detect cell cycle alterations in primary cortical cultures [30,42]. In this work, we therefore focused on delineating signal transduction pathways by Western blotting, coupled with immunocytochemical colocalization of cyclin D1 in the MAP-2^+^ neurons (Figure 2B,C) and BrdU incorporation (Figure 2G,H), without comprehensively conducting all these experiments to re-affirm neuronal CCR in primary cortical cultures.

In addition to expression levels, other mechanisms may also contribute to activation of PKCδ. For example, PKCδ may be proteolytically activated by caspase-3, which is crucial for apoptosis induced by 1-methyl-4-phenylpyridinium (MPP^+^), a pharmacological model mimicking Parkinson’s disease (PD). In rat dopaminergic neurons, exposure to MPP^+^ time-dependently increases caspase-3 activity. Notably, MPP^+^ causes the cleavage of the approximately 74-kDa PKCδ into a 41-kDa catalytic subunit and a 38-kDa regulatory subunit with a sustained increase in its kinase activity; the caspase-3 inhibitor Z-DEVD-fmk effectively blocks MPP^+^-induced PKCδ cleavage and kinase activity, suggesting that caspase-3 acts upstream of proteolytic activation of PKCδ [43]. In this work, we reported that PKCδ induced by Aβ25-35/1-42 triggered caspase-3 cleavage (Figure 2F and Figure 5D), suggesting that caspase-3 is downstream of PKCδ. Whether this caspase-3 activation constitutes a positive feedforward mechanism to trigger further activation of PKCδ in our experimental system requires further investigation. In another study, PKCδ is shown to be a kinase sensitive to oxidative stress. Exposure of N27, a mesencephalic dopaminergic neuronal cell line, to H_2_O_2_ dose-dependently triggers caspase-3 activation and PKCδ cleavage; H_2_O_2_ also increases phosphorylation of PKCδ on Tyr-311 [44]. Notably, it has been shown that H_2_O_2_ mediates Aβ neurotoxicity [45]. These findings demonstrate that caspase-3 and oxidative stress can regulate activation of PKCδ by tyrosine phosphorylation. However, whether Aβs directly triggers activation of PKCδ via Tyr-311 phosphorylation still remains unknown and awaits further studies.

In this work, pharmacological activation of PKCδ was achieved by using PMA (Figure 3 and Figure 4), which is known to activate other PKC isoforms such as PKCα, β, γ, ε, η, and θ [46]. Therefore, our experimental results derived from using this compound should be interpreted with caution, as PMA may also activate other PKC isoforms, along with induction of relevant signaling pathways in the post-mitotic neurons. However, given that lentivirus-mediated expression of PKCδ shRNA, which completely knocked down its expression (Figure 1B), also significantly suppressed PMA-dependent neuronal CCR and apoptosis (Figure 3) as well as p35 cleavage into p25 (Figure 4D), it appears to be less likely that the observed PMA effects were due to activation of other PKC isoforms independent of PKCδ. Conceivably, a direct approach to address this issue is to overexpress the full-length PKCδ cDNA into cortical neurons. Unfortunately, the 74-kDa molecular weight of PKCδ makes it difficult, if not entirely impossible, for lentivirus-mediated overexpression in primary cortical neurons; furthermore, forced expression of PKCδ may potentially cause neurotoxicity. Since the present work mainly focused on whether PKCδ induced by Aβs is necessary for its neurotoxicity, we did not pursue overexpression of PKCδ in primary neurons further to test whether this gene manipulation alone is sufficient to trigger neuronal CCR.

The correlation between PKCδ and CDK5 in the nervous system has not been clearly delineated. PKCδ can phosphorylate p35 to attenuate its degradation, thus regulating p35/CDK5 activity [47]. PKCs may indirectly control CDK5 activity through regulating its neuronal cofactor in the brain [48]. In our previous study, we also reported that CDK5/HIF-1 contributes to Aβ-induced neuronal CCR and caspase-dependent apoptosis in post-mitotic neurons [30]. Consistent with these findings, we observed that PKCδ-dependent p35 cleavage, CCR, and consequent apoptosis induced by Aβ25-35 are mitigated by inhibition of calpain2, but not calpain1 (Figure 7). This result is consistent with the previous report that polybrominated diphenyl ethers induce neuronal apoptosis through calpain2-, but not calpain1-dependent cleavage of p35 into p25, with resultant overactivation of CDK5 [49]. Unexpectedly, we found that knockdown of PKCδ by shRNA induced the expression of calpain1 with or without Aβ25-35 exposure (Figure 7F), implying a negative impact of PKCδ on the expression of endogenous calpain1. Since caplain1 was not involved in the Aβ25-35 effects on neuronal CCR and apoptosis, in this work we did not further pursue the detailed underlying mechanisms in order to stay focused.

Downstream of CDK5, we observed the induction of PUMA, a pro-apoptotic mediator, which was also dependent on PKCδ (Figure 8A,B). One earlier study reported that DNA damage may stimulate CDK5 to directly phosphorylate ATM (ataxia-telangiectasia mutated) in post-mitotic neurons, leading to p53 activation; notably, disruption of the CDK5-ATM cascade lessens DNA-damage-induced neuronal CCR and expression of PUMA and the pro-apoptotic protein Bax, thereby protecting neurons from death [50,51]. In another study, inhibition of CDK5 activity also attenuated p53-dependent apoptosis of hippocampal CA1 pyramidal neurons following cerebral ischemia; further, after transient global ischemia, the increased expression levels of Bax, PUMA, and activated caspase-3 were all decreased by roscovitine, a CDK5 inhibitor [52]. Our results are consistent with these previous findings that p53/PUMA may play a pivotal role in CDK5-dependent neuronal CCR and apoptosis. We further reveal the novel finding that PKCδ may induce PUMA in neurons via CDK5, thereby leading to neuronal apoptosis.

The correlation between STAT3 and PKCδ in the nervous system has been less well understood. One report demonstrated that tumor necrosis factor-like weak inducer of apoptosis (TWEAK)-induced PKCδ enhances STAT3 activation, along with production of proinflammatory mediators in astrocytes, indicating that PKCδ is an upstream regulator of STAT3; further, these findings can be recapitulated in the MPTP (1-methyl-4-phenyl-1,2,3,6-tetrahydropyridine) mouse model of PD [53]. In this work, we found that STAT3 shRNA markedly attenuated Aβ25-35-induced PKCδ expression, p35 cleavage into p25, expression of the S-phase marker PCNA, and caspase3 cleavage in post-mitotic neurons (Figure 6E–H), suggesting STAT3 acts upstream of PKCδ. The rationale underlying these inconsistent findings remains unknown. One possibility is that differentiated cortical neurons, but not astrocytes, were used as the in vitro model system in the present study. Moreover, since we did not test whether PKCδ shRNA reciprocally suppresses STAT3 expression, such a possibility cannot be completely excluded.

While STAT3 is well known to be implicated in the regulation of cell cycle progression in tumor cells, its involvements in cell cycle reactivation and neuronal apoptosis in the central nervous system are less well understood. Earlier it was reported that heightened tyrosine phosphorylation of STAT3 was detected in the cortex and hippocampus of APP/PS1 transgenic mice. Exposure of cultured neurons to Aβ and intrahippocampal injection of Aβ into mouse brains were sufficient to induce STAT3 phosphorylation, wherein Tyk2 was identified as the tyrosine kinase that acts upstream of STAT3 [54]. Consistent with these findings, in this study we observed that Aβ25-35 triggered the induction of STAT3 and, more importantly, the PKCδ/calpain2/p25-CDK5 signaling acted downstream of STAT3 and contributed to neuronal CCR, with subsequent caspase-3 cleavage in the post-mitotic neurons. However, the detailed mechanisms underlying the observed Aβ induction of PKCδ via STAT3 remain unclear and need to be further studied. Another more recent work reported that remote ischemic pre-conditioning (RIPC) exerts protective effects against brain injury resulted from ischemia/reperfusion; interestingly, the salutary effects of RIPC involve downregulation of the STAT3 pathway, with resultant inhibition of neuronal CCR by inhibiting cyclin D1 and CDK6 triggered by transient focal ischemia [55]. Herein, we report the critical role of CDK5 mediating Aβ-induced neuronal CCR and apoptosis. Whether CDK6 also contributes to the observed Aβ effects in our experimental model system requires further investigation. 

The present study was conducted with in vitro differentiated primary rat cortical neurons; the clinical relevance of our findings thus remains to be further confirmed using the AD transgenic mouse model or even clinical specimens. Nevertheless, several previous reports have indicated that these pro-apoptotic mediators triggered by Aβs, including PKCδ, CDK5, and STAT3, may serve as potential biomarkers in plasma or cerebrospinal fluid (CSF) for AD in clinical settings. First, it was recently demonstrated that PKCδ levels were dramatically increased in the CSF of AD patients and positively correlated with cytokines; similar findings were observed in AD transgenic mice. Mechanistically, the secretion of PKCδ from microglia can be stimulated by Aβ, thus leading to upregulation of the NF-κB pathway, along with overproduction of proinflammatory cytokines; the authors therefore suggested that PKCδ may serve as a potential biomarker and therapeutic target for microglia-mediated neuroinflammation in AD [37]. Second, one study that performed the proteomic analysis of CSF revealed a decrease in seven CSF proteins in the AD patients; among these, four carry neuroprotective actions, while the remaining three proteins, including CDK5, promote neuronal death [56], suggesting that CDK5 may be considered a biomarker for AD, at least in CSF. In addition to serving as a direct biomarker, CDK5 has been shown to be correlated with other biomarkers in AD. For example, one study investigated the intercorrelation among long non-coding RNA MALAT1 (lnc-MALAT1), microRNA-125b (miR-125b), and their targets including CDK5 as well as their correlations with disease severity of AD [57]. It was found that CSF miR-125b/CDK5 levels were upregulated in AD patients compared with PD patients and controls; further, CSF/plasma miR-125b positively correlated with CDK5. Notably, miR-125b and CDK5 correlated with exacerbated disease severity as manifested by Aβ42, total tau, and phosphorylated tau, as well as the mini-mental state examination (MMSE) score in AD patients, but not in PD patients or controls. The authors therefore concluded that lnc-MALAT1 and its target miR-125b are potential biomarkers for AD management via interaction with their targets, including CDK5 [57]. Third, STAT3 has not been extensively reported to serve as a biomarker for AD in clinical studies. Nevertheless, one report has revealed that platelet activating factor receptor (PTAFR), which is significantly upregulated in the brain tissue, peripheral blood, and CSF of AD patients, may be a potential AD biomarker; notably, PTAFR triggers the inflammatory responses mediated by microglia through the interleukin (IL)-10/STAT3 pathway [58]. Together, these pro-apoptotic mediators induced by Aβ may either by themselves or indirectly serve as potential biomarkers for early diagnosis or evaluating the therapeutic efficacies of intervention strategies for AD in clinical settings.

In this work, we presented in vitro evidence that hyperactive CDK5 mediates Aβ neurotoxicity in post-mitotic neurons. Whether *CDK5* gene polymorphisms are a risk factor for AD requires further investigation, as conflicting findings were observed. One report suggested that *CDK5* gene polymorphism may be associated with the risk of AD in a Dutch population-based study, because a significantly increased risk for carriers of the GG genotype of a single nucleotide polymorphism (SNP), rs2069442, was detected in those AD patients without the APOEε4 allele [59]. Another study examined several genetic variations of the *CDK5* gene (rs9278, rs2069459, rs891507, rs2069454, rs1549759, and rs2069442) in Spanish AD cases and controls; however, no differences in the genotypic, allelic, or haplotypic distributions between AD cases and controls were detected [60]. It should be noted that these genetic variants of the *CDK5* gene in the Spanish AD cases included the SNP rs2069442 that has been associated with AD risk in the Dutch population [59]. One additional study investigated the influence of rs2069456 SNP (A→C, intron 7) in the *CDK5* gene on the risk of AD, as well as the biochemical and neuropathological markers. However, despite the increased levels of total tau and phosphorylated tau and the decreased levels of Aβ1-42 in the CSF derived from AD patients compared with the control group, total tau, phosphorylated tau, and Aβ1-42 levels in the CSF were not influenced by this rs2069456 SNP in the *CDK5* gene [61]. Obviously, more studies are required to reveal the genetic variants of the CDK5 gene for predicting the risk of AD in different populations.

In conclusion, we demonstrated that, in the post-mitotic neurons exposed to Aβs, STAT3-induced PKCδ regulates p35 cleavage into p25 via calpain2, thereby inducing expression of proapoptotic protein PUMA, aberrant cell cycle reactivation, and consequent apoptosis. Our experimental evidence thus supports the signaling cascade of “Aβ → STAT3 → PKCδ → calpain2 → CDK5/p25 → CCR/PUMA/caspase-3 → apoptosis” in differentiated post-mitotic cortical neurons (Figure 9). PKCδ inhibition mitigates cytotoxicity and restores neuronal damages induced by Aβs in vitro, suggesting that PKCδ inhibition may represent a potential therapeutic target for AD.

## 4. Materials and Methods

### 4.1. Reagents, Preparations of Aβs, and Primary Culture of Rat Cortical Neurons

PMA (Cat. No. P1585, Sigma-Aldrich, St. Louis, MO, USA) was dissolved in dimethyl sulfoxide (DMSO) to make a stock solution of 5 mg/ml; the final working concentration was 40 ng/ml in culture medium. 6-Nitro-1-benzothiophene 1,1-dioxide (Stattic; Cat. No. ab120952, Abcam, London, UK) was dissolved in DMSO to make a stock solution of 50 mM; the final working concentration was 2.5 μM in culture medium. Aβ25-35 (Cat. No. A4559, Sigma-Aldrich), Aβ1-42 (Cat. No. A-1163-2; rPeptide, Watkinsville, GA, USA), and BrdU (Cat. No. 550891; BD Biosciences, San Jose, CA, USA) were prepared essentially as described in our previous report [32]. All the procedures for animal care and preparation of fetal rat cortical cultures were approved by the Institutional Animal Care and Use Committee (IACUC) of National Yang Ming Chiao Tung University (No. 1110434). Cortical neurons were cultured from fetal (embryonic day 18) brains of Sprague–Dawley (SD) rats as described previously [62]. The neurons were kept in a humidified incubator at 37 °C with 5% CO_2_ for at least 7 days to allow differentiation and used during 7–10 days in vitro (DIV).

### 4.2. Western Blotting

Western blotting was conducted as described in our previous publications [30,32]. The rabbit antibodies against total caspase-3 (1:1000; Cat. No. 9662, Cell Signaling, Danvers, MA, USA), CDK5 (1:1000; Cat. No. ab40773; Abcam), p35/p25 (1:1000; Cat. No. 2680, Cell Signaling), and calpain2 (1:1000; Cat. No. 2539, Cell Signaling) as well as the mouse antibody against α-tubulin (1:5000; Cat. No. SI-T9026, Sigma-Aldrich) and β-actin (1:5000; Cat. No. MAB1501, Sigma-Aldrich) were all diluted in blocking buffer (5% nonfat dry milk in PBST buffer containing 0.05% Tween-20). The rabbit antibodies against protein kinase Cδ (PKCδ; 1:1000; Cat. No. 9616, Cell Signaling), cyclin D1 (1:1000; Cat. No. ab134175, Abcam), PCNA (1:1000; Cat. No. ab92552, Abcam), p-Histone H3 (1:1000; Cat. No. ab32107, Abcam), cleaved caspase-3 (1:1000; Cat. No. 9664, Cell Signaling), STAT3 (1:1000; Cat. No. 12640, Cell Signaling), calpain1 (1:1000; Cat. No. 2556, Cell Signaling), and p53-upregulated modulator of apoptosis (PUMA; 1:1000; Cat. No. ab9643, Abcam) were all diluted in signal enhancer HIKARI solution 1 (Cat. No. NT08044-71R, Nacalai Tesque, Kyoto, Japan). All the hybridizations with the primary antibodies were at 4 °C overnight. Following hybridization with the corresponding horseradish peroxidase (HRP)-conjugated secondary antibodies at 1:5000, the immunoreactive signals were detected using ECL-Plus Western blotting detection reagents (Cat. No. FL0010-0125, Bionovas, Toronto, Canada). The blots were exposed under a Luminescence Imaging System (Amersham Imager 600, FUJIFILM, Tokyo, Japan) and the signal intensity was quantified by ImageJ software (Version 1.44p, National Institutes of Health, Bethesda, MD, USA).

### 4.3. Lentiviral Infection of shRNA in Primary Rat Cortical Cultures

The timeline for lentivirus-mediated knockdown of gene expression can be found in the supplementary file entitled “Figure S1-PKCdelta and Abeta-Full Blots and Workflow for Lentivirus Infection”. The lentiviral vectors and shRNA plasmids were produced by RNAiCore (Nankang, Taipei, Taiwan). To knock down the target proteins, primary cortical neurons were transfected with the target shRNA or the negative control shRNA via lentiviral infection at a multiplicity of infection (MOI) of 2 for 1 d (DIV4-5) in NB/B27 medium. The virus-containing medium was then replaced by the mixed medium containing equal volumes of fresh NB/B27 medium and the “old” NB/B27 medium, which had been used to culture these same neurons for 4 d during DIV0-4, before further experimentation. The target sequences from RNAiCore shRNA lentivirus are as follows: 5′-GCTGGGAGTAACAGGAAACAT-3′ for PKCδ, 5′-CGGGAGATCTGTCTACTCAAA-3′ for CDK5, 5′-CCTGAGTTGAATTATCAGCTT-3′ for STAT3, 5′-GCGGTCAGATACCTTCATTAA-3′ for calpain2, and 5′-GCCGTGGACTTTGACAACTTT-3′ for calpain1. The sequence of the non-targeting shRNA used as a negative control in all shRNA experiments is as follows: 5′-CGCGATCGTAATCACCCGAGT-3′ for shLac and 5′-GCGGTTGCCAAGAGGTTCCAT-3′ for shLuc.

### 4.4. Cell Survival Assays

The PI/Hoechst double staining to assess the extents of cell survival was conducted as described in our previous report [63]. “Death index (%)” was defined as the number of PI-positive nuclei, which represented the dying or dead cells, divided by that of Hoechst-positive nuclei in each vision field. The MTT reduction assay was performed according to our earlier study [64].

### 4.5. Immunocytochemistry and Quantification of Neurite Lengths and Neurite Branches

Immunocytochemistry was performed as described in our previous publications [30,32]. The same rabbit antibody against cyclin D1 (1:100) used in Western blotting and the Alexa Fluor 488-labeled goat anti-rabbit IgG (1:500; Cat. No. A11034; Invitrogen) were used to label the G1-phase cells. The mouse monoclonal antibody against MAP-2 (1:100; Cat. No. MAB378, CHEMICON International, Temecula, CA, USA) and the secondary antibody, Hilyte Fluor 594-labeled goat anti-mouse IgG (1:200; Cat. No. 61507-H594, AnaSpec, Fremont, CA, USA), were used to stain the mature neurons. Detection of BrdU in NeuN-positive neurons by immunocytochemistry was performed as described [32]. In brief, a rat primary antibody against BrdU (1:200; Cat. No. 6326, Abcam) and fluorescein isothiocyanate (FITC)-conjugated rabbit anti-rat IgG (1:100; Cat. No. ab6730, Abcam) were used for detection of BrdU^+^ cells. The mouse monoclonal antibody against NeuN (1:100; Cat. No. ABN78, CHEMICON/Millipore Corp.) was applied to stain the mature neurons. For confocal microscopy, the samples were observed under a laser-scanning confocal microscope (Zeiss LSM700, Oberkochen, Germany) equipped with filter sets to detect the corresponding fluorescence signals. The neurite lengths and neurite branches are two indices for neuronal growth and differentiation, which were quantified using MetaMorph software (Version 7.7, Molecular Devices, LLC., San Jose, CA, USA). A series of analyses were conducted on both the blue channel (Hoechst staining for nuclei) and the red channel (MAP-2 staining for mature neurons) to quantitatively determine their respective signals. The nuclei were detected by setting the width, area, and background ranges. Neurites were selected within ranges of the neurite width and signal intensity, which were greater than a user-defined minimum value above a defined background.

### 4.6. Statistical Analysis

Results are expressed as mean ± S.E.M. from the sample number (N). Each N represents the data derived from one independent experiment using one different culture. Data were analyzed by one-way analysis of variance (ANOVA), followed by a post hoc Tukey test. *p*-values of less than 0.05 are considered significant.

## Data Availability

All data generated or analyzed during this study are included in this published article.

## Supplementary Materials

The following supporting information can be downloaded at: https://www.mdpi.com/article/10.3390/ijms25179626/s1.
