# Supplementary material for: Protein Kinase C-Delta Mediates Cell Cycle Reentry and Apoptosis Induced by Amyloid-Beta Peptide in Post-Mitotic Cortical Neurons
Source: Int J Mol Sci. 2024 Sep 5;25(17):9626. doi: 10.3390/ijms25179626 (PMC11395613; doi:10.3390/ijms25179626)
Supplement: Supplementary file 1 [file ijms-25-09626-s001.zip › ijms-3174818-supplementary.pdf]

**Title:** Protein kinase C-delta mediates cell cycle reentry and apoptosis induced by amyloid-beta peptide in post-mitotic cortical neurons

**Authors**: Ming-Hsuan Wu, A-Ching Chao, Yi-Heng Hsieh, You Lien, Yi-Chun Lin, and Ding-I Yang
**Affiliation and E-mail**: Institute of Brain Science, National Yang Ming Chiao Tung University, Taipei 112304, Taiwan; diyang@nycu.edu.tw

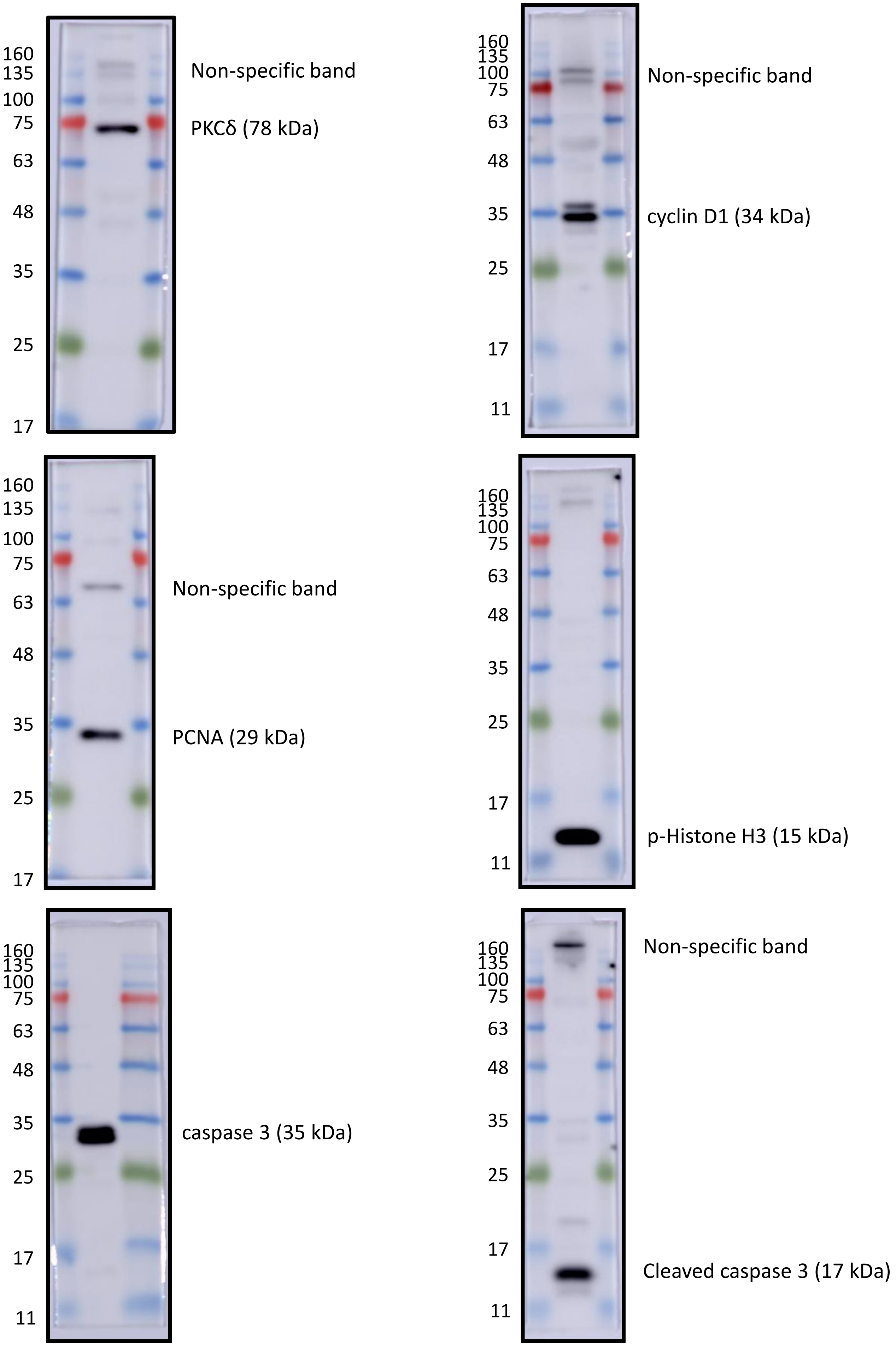
160
135
100
75
63
48
35
25
17
Non-specific band
PKCδ (78 kDa)
160
135
100
75
63
48
35
25
17
11
Non-specific band
cyclin D1 (34 kDa)
160
135
100
75
63
48
35
25
17
Non-specific band
PCNA (29 kDa)
160
135
100
75
63
48
35
25
17
11
p-Histone H3 (15 kDa)
160
135
100
75
63
48
35
25
17
11
caspase 3 (35 kDa)
160
135
100
75
63
48
35
25
17
11
Non-specific band
Cleaved caspase 3 (17 kDa)

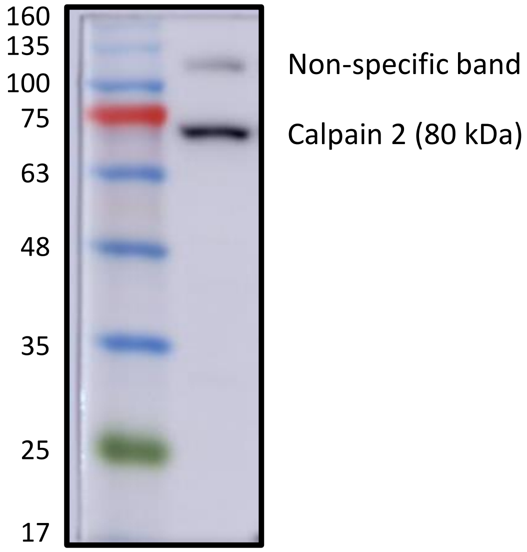
160
135
100
75
63
48
35
25
17
Non-specific band
Calpain 2 (80 kDa)

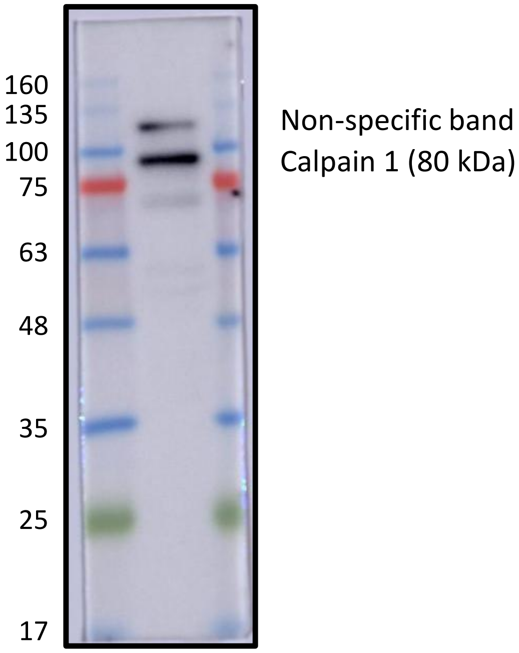
160
135
100
75
63
48
35
25
17
Non-specific band
Calpain 1 (80 kDa)

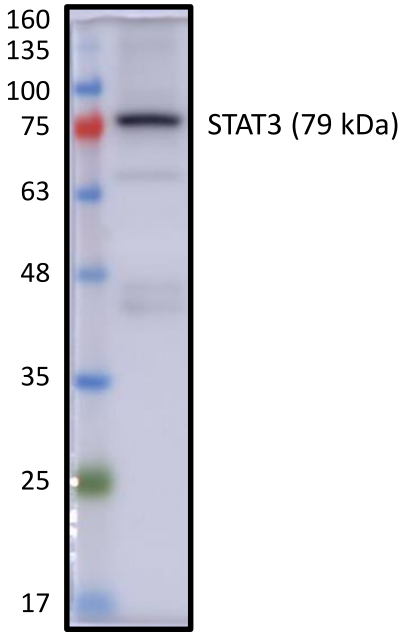
160
135
100
75
63
48
35
25
17
STAT3 (79 kDa)

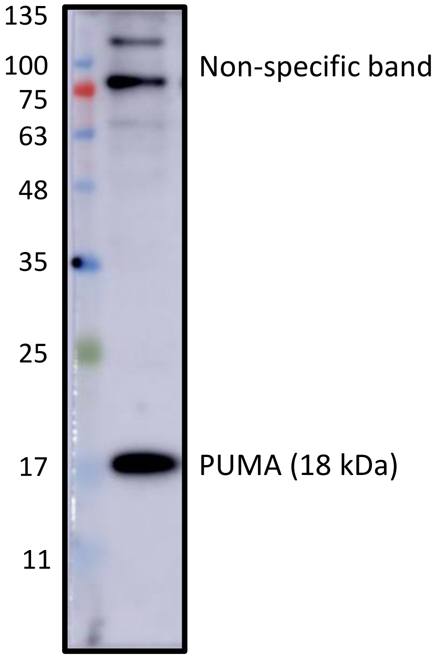
135
100
75
63
48
35
25
17
11
Non-specific band
PUMA (18 kDa)

## Figure 1A

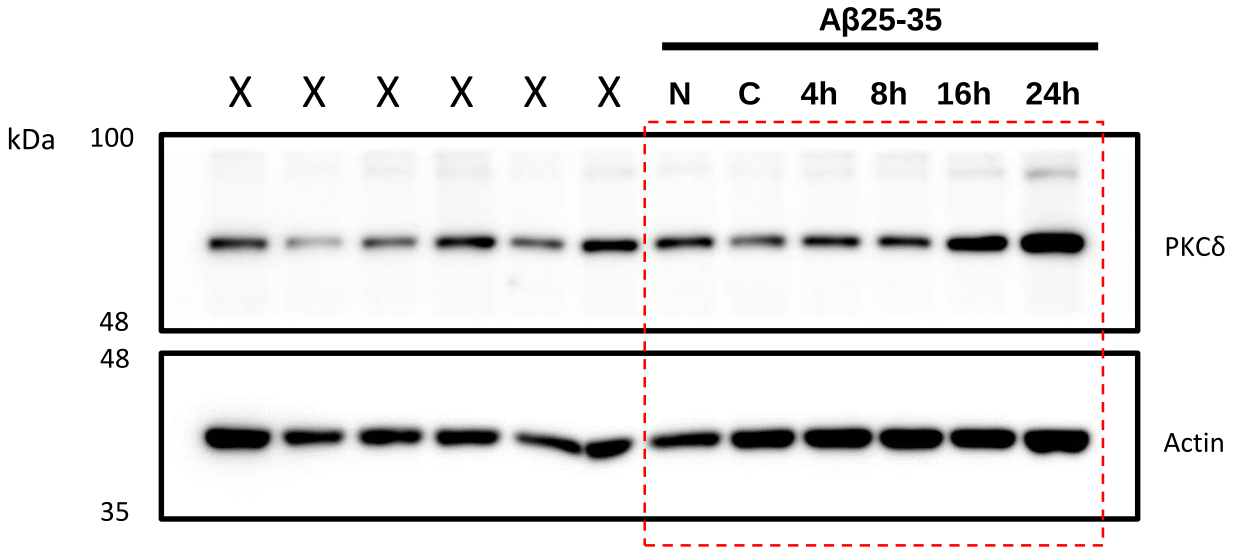


Right six lanes are shown in the figure; left six lanes are for other experiments.

## Figure 1B

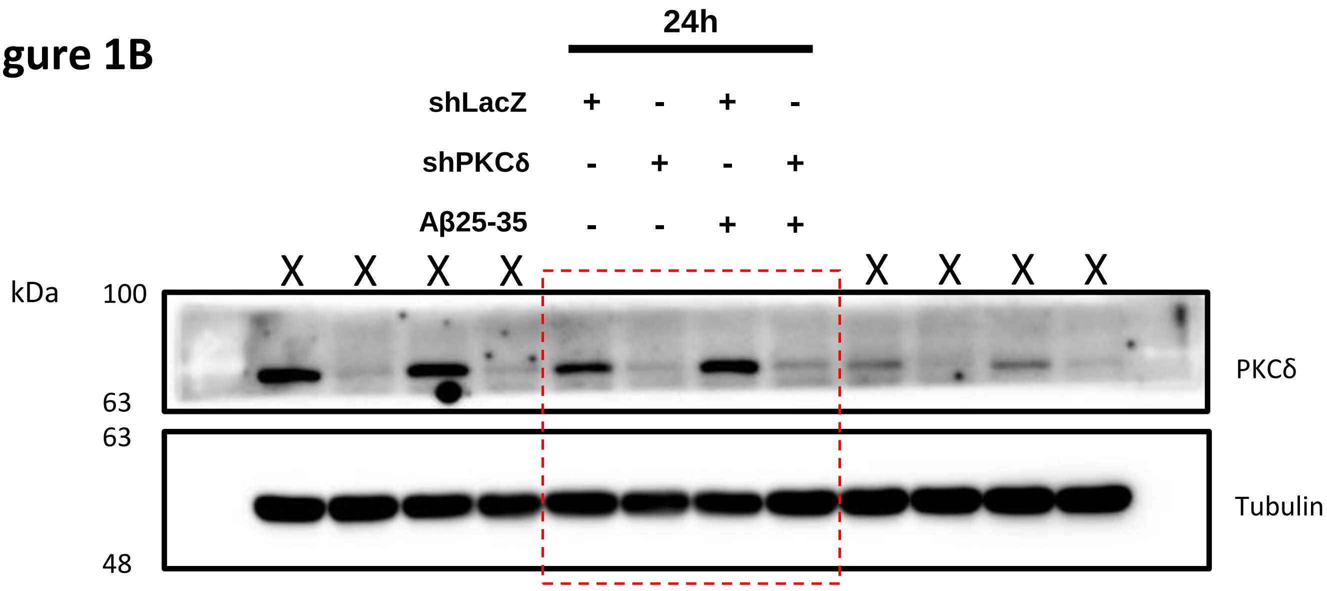


Middle four lanes are shown in the figure; left and right four lanes are for other experiments.

# Figure 2A

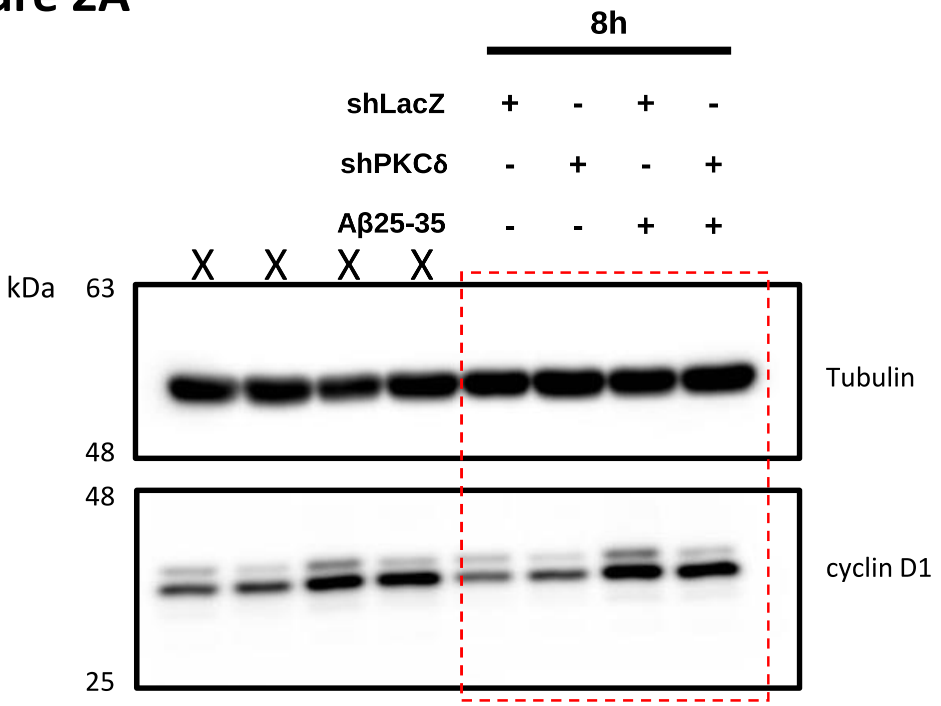


Right four lanes are shown in the figure; left four lanes are for other experiments.

# Figure 2D

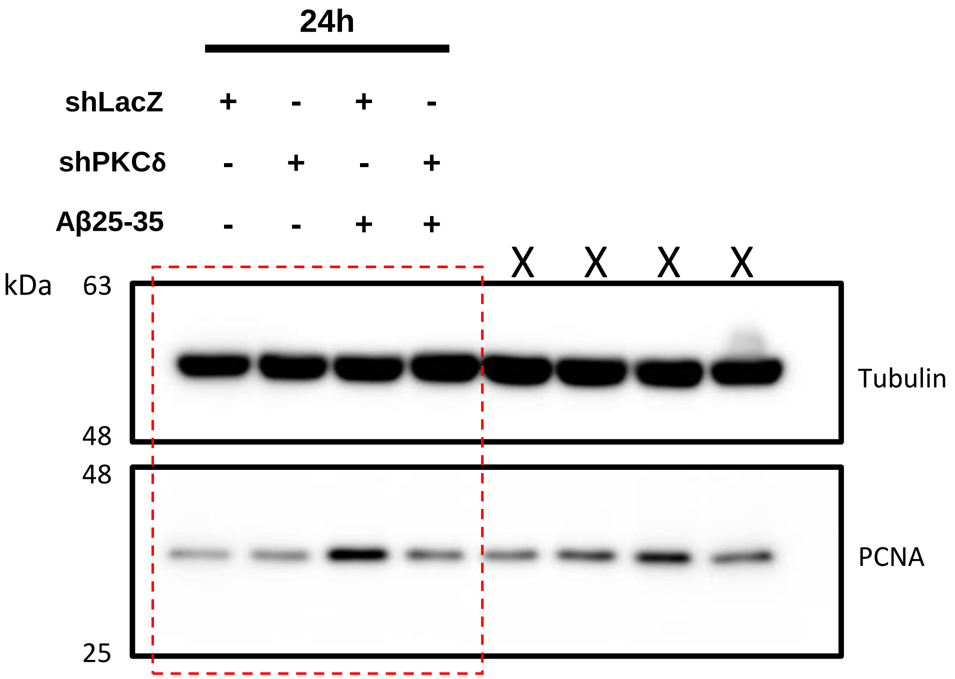


Left four lanes are shown in the figure; right four lanes are for other experiments.

# Figure 2E

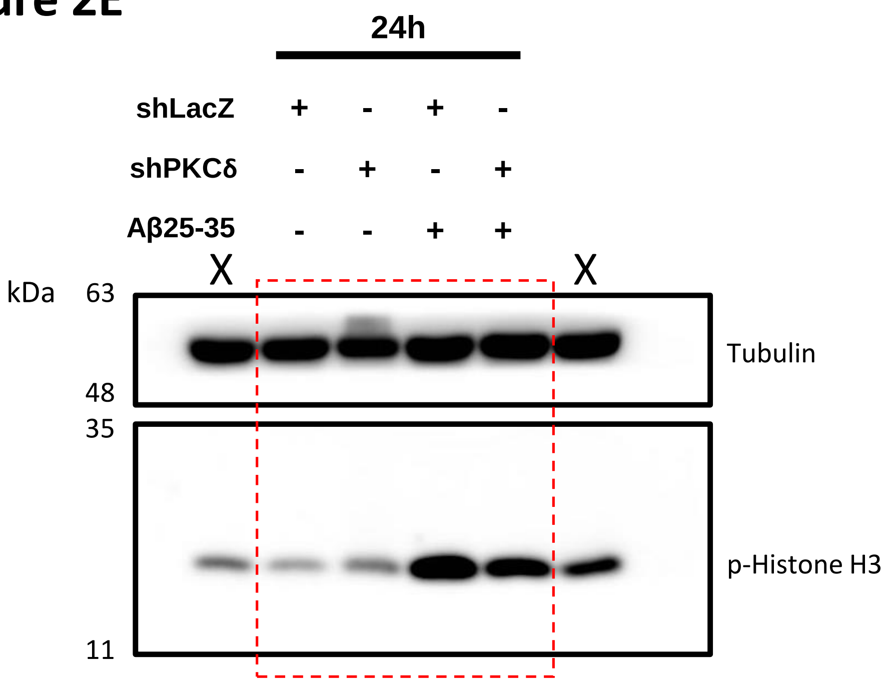


Middle four lanes are shown in the figure; left and right lanes are for other experiments.

# Figure 2F

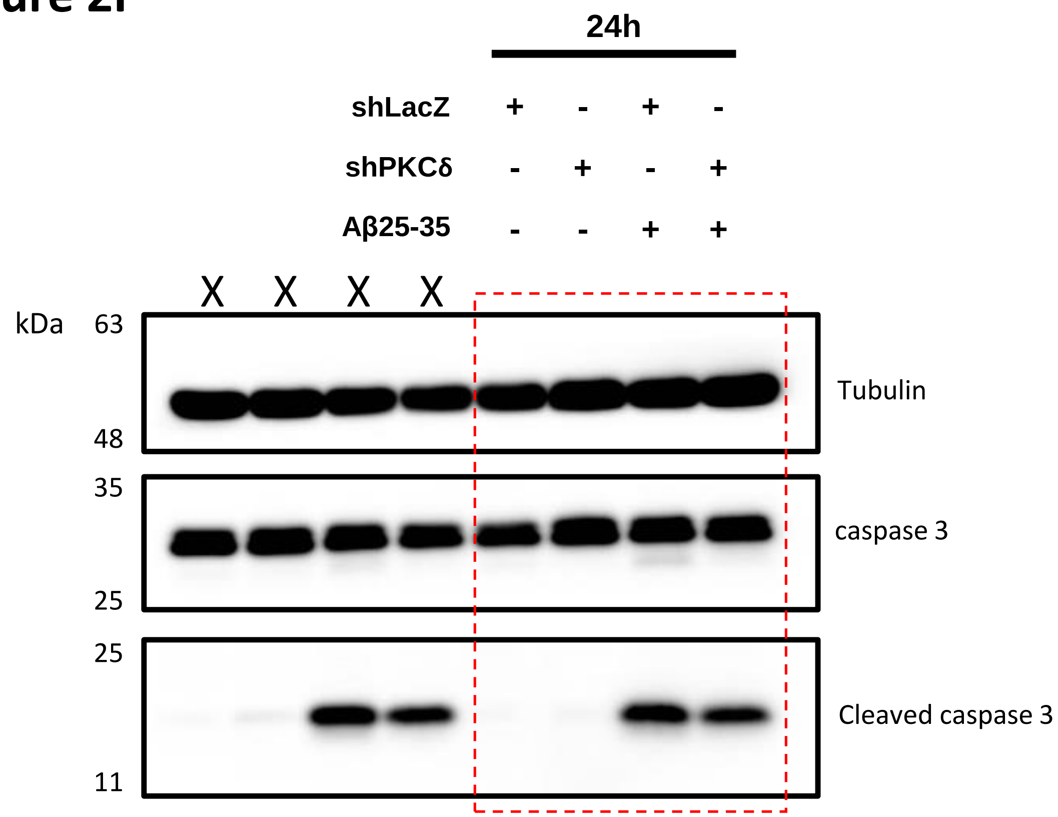


Right four lanes are shown in the figure; left four lanes are for other experiments.

## Figure 3A

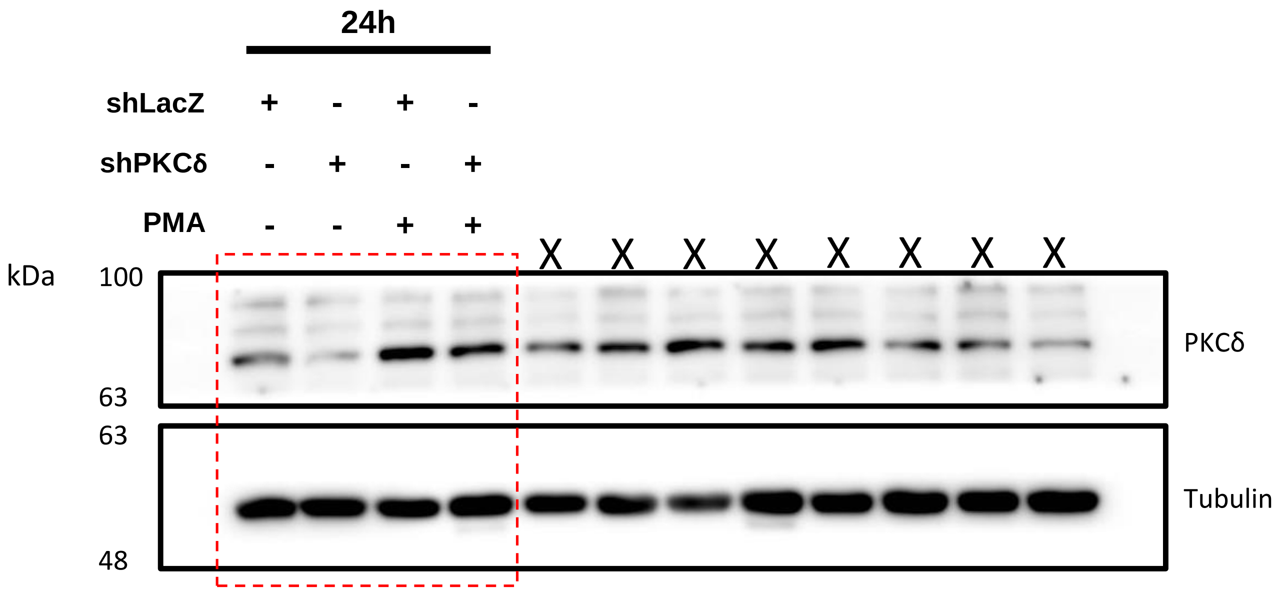


Left four lanes are shown in the figure; right eight lanes are for other experiments.

## Figure 3B

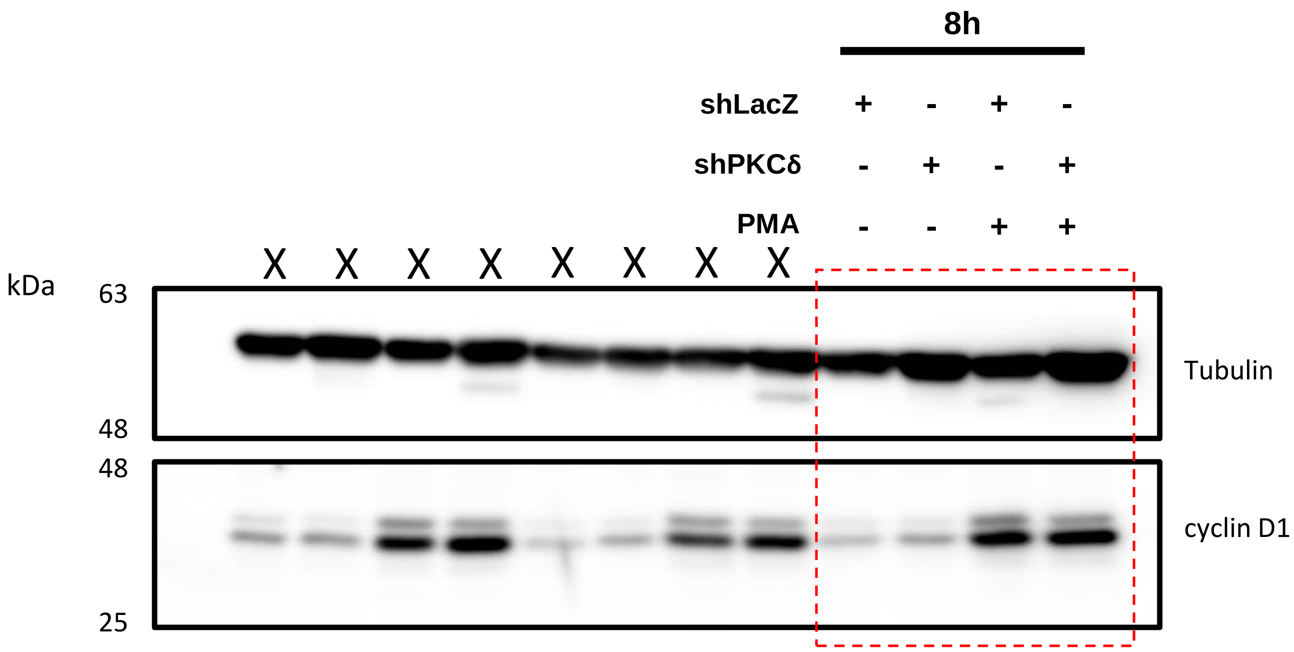


Right four lanes are shown in the figure; left eight lanes are for other experiments.

## Figure 3C

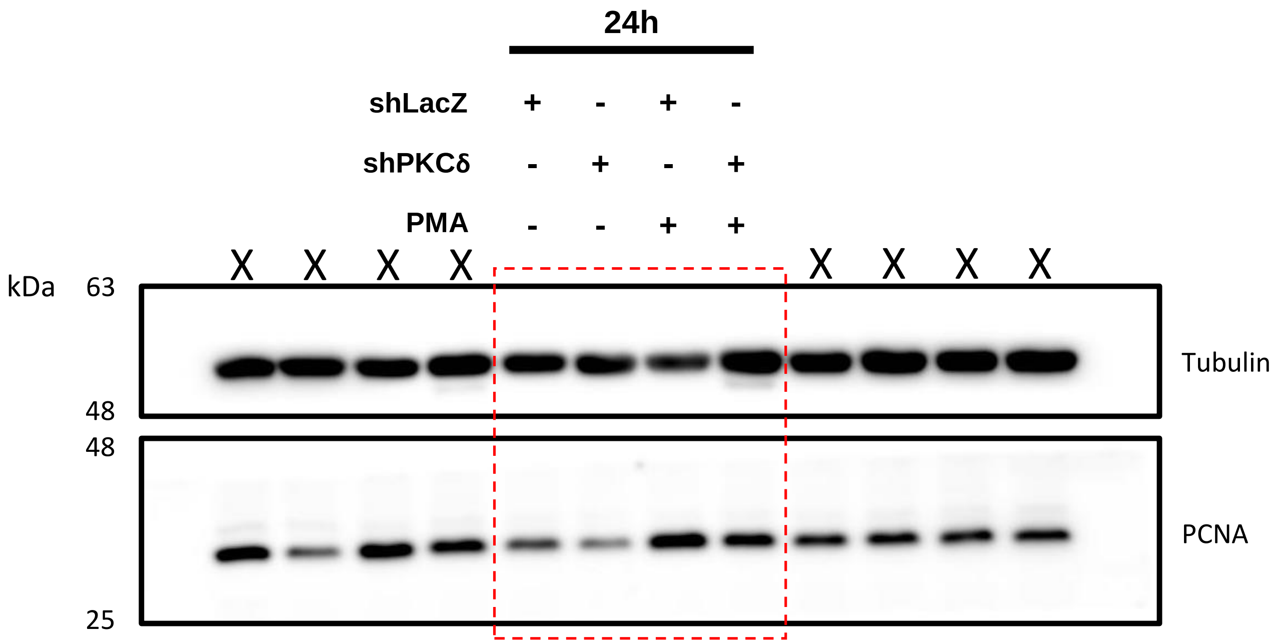


Middle four lanes are shown in the figure; left and right four lanes are for other experiments.

## Figure 3D

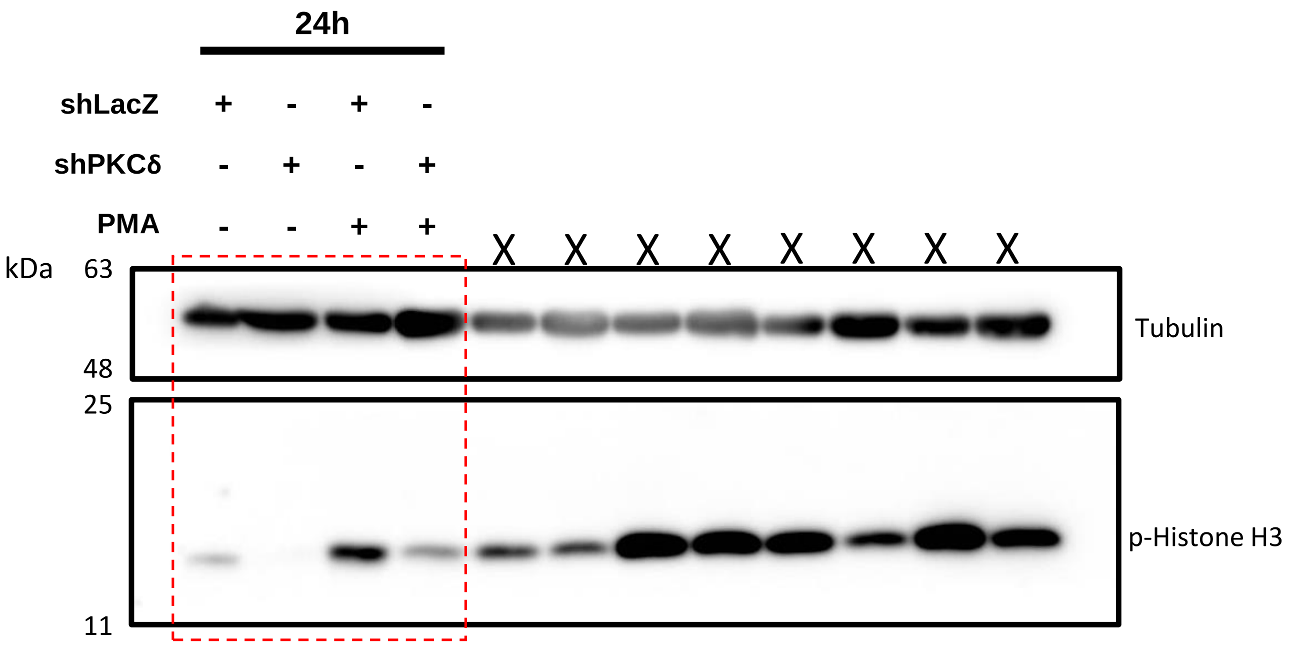


Left four lanes are shown in the figure; right eight lanes are for other experiments.

# Figure 3E

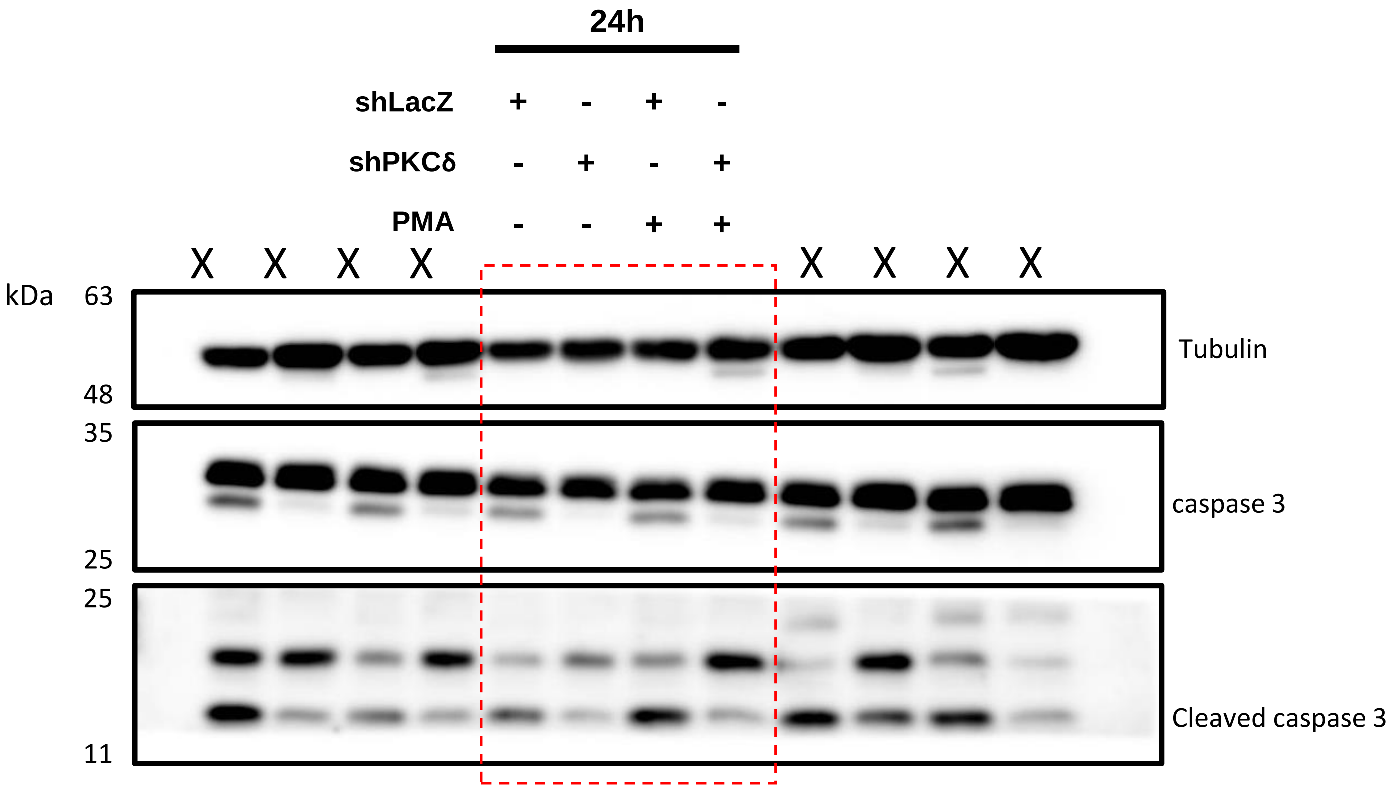


Middle four lanes are shown in the figure; left and right four lanes are for other experiments.

# Figure 4A

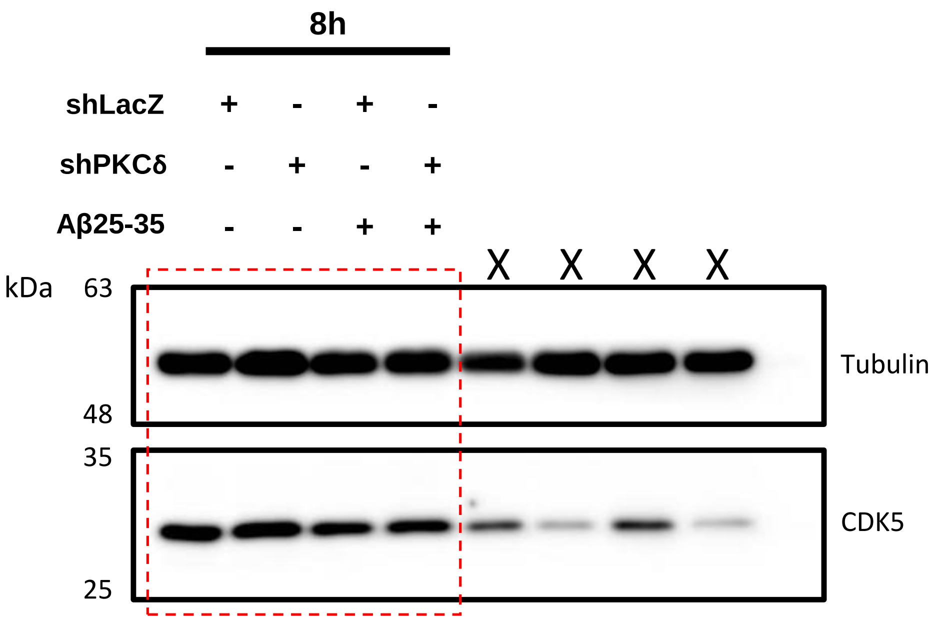


Left four lanes are shown in the figure; right four lanes are for other experiments.

# Figure 4B

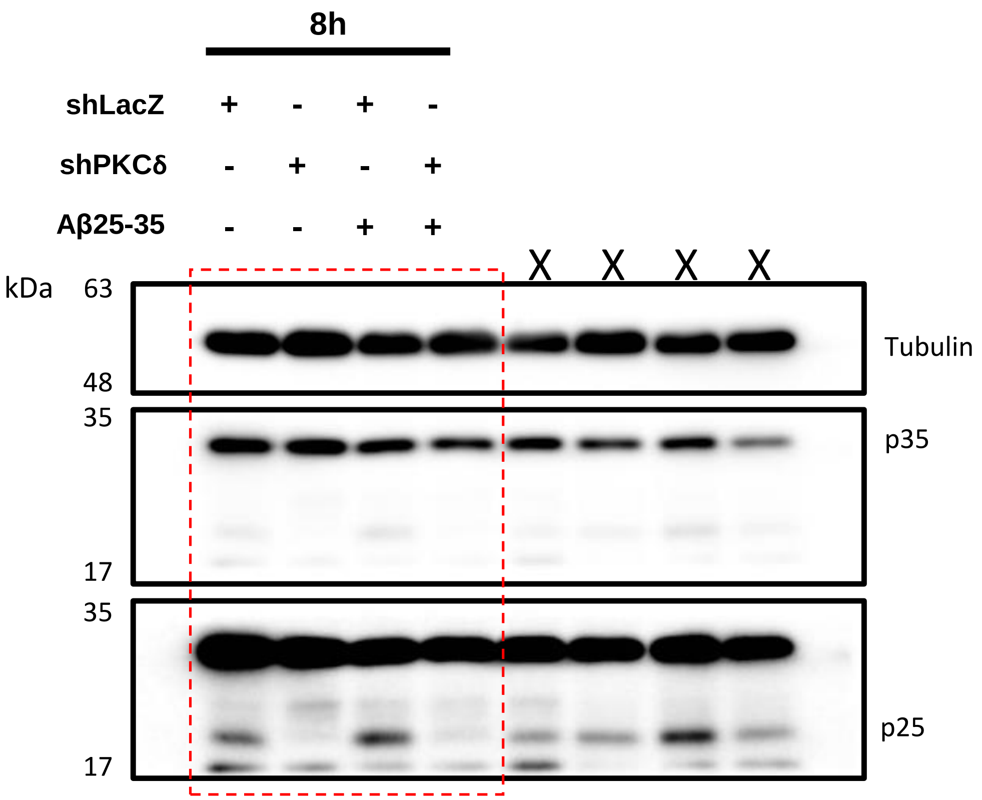


Left four lanes are shown in the figure; right four lanes are for other experiments.
Note that results of p35 and p25 are same membrane except having different exposure time.

## Figure 4C

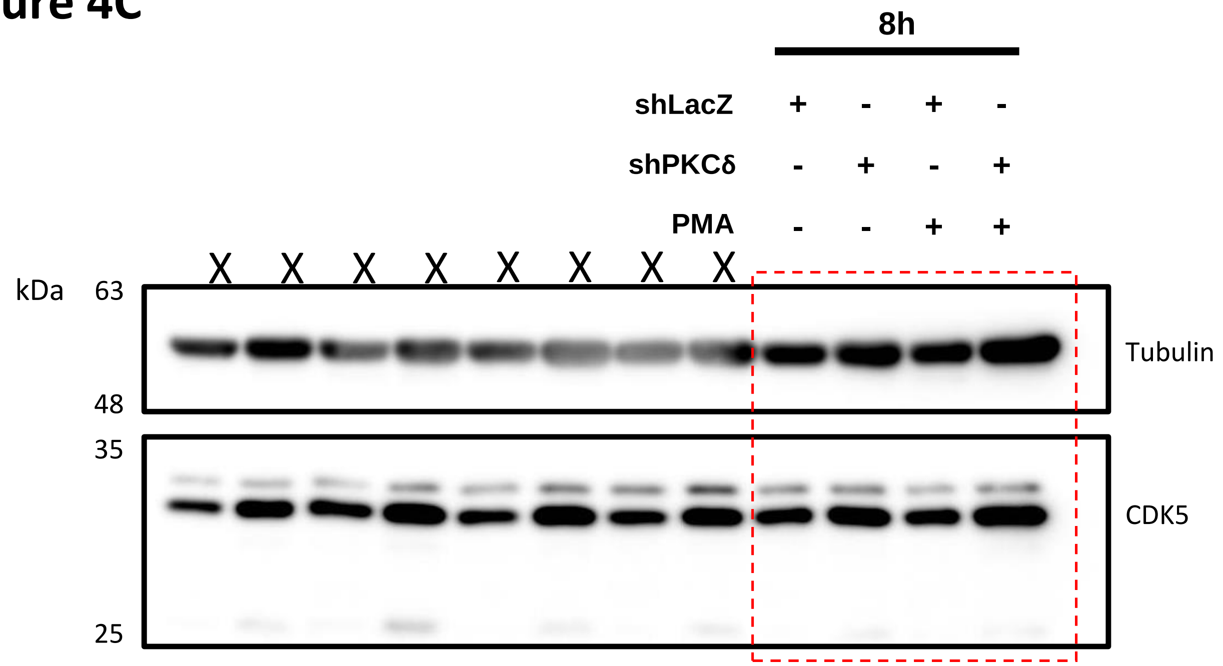


Right four lanes are shown in the figure; left eight lanes are for other experiments.

## Figure 4D

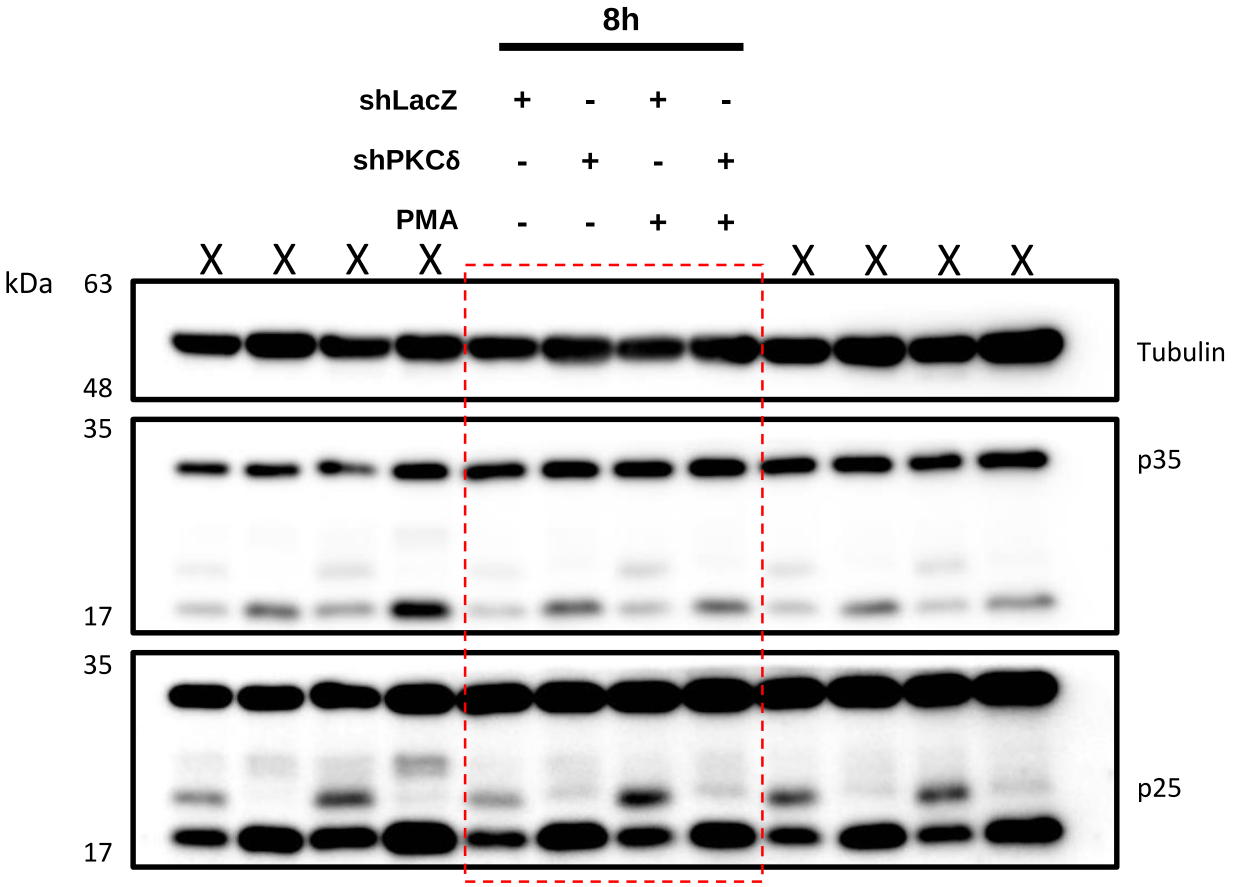


Middle four lanes are shown in the figure; left and right four lanes are for other experiments.

# Figure 4E

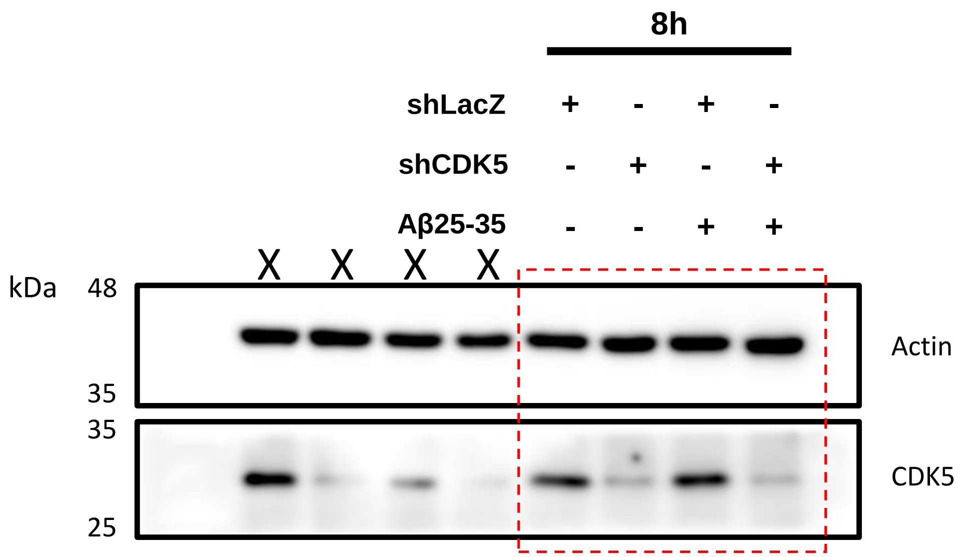


Right four lanes are shown in the figure; left four lanes are for other experiments.

# Figure 4F

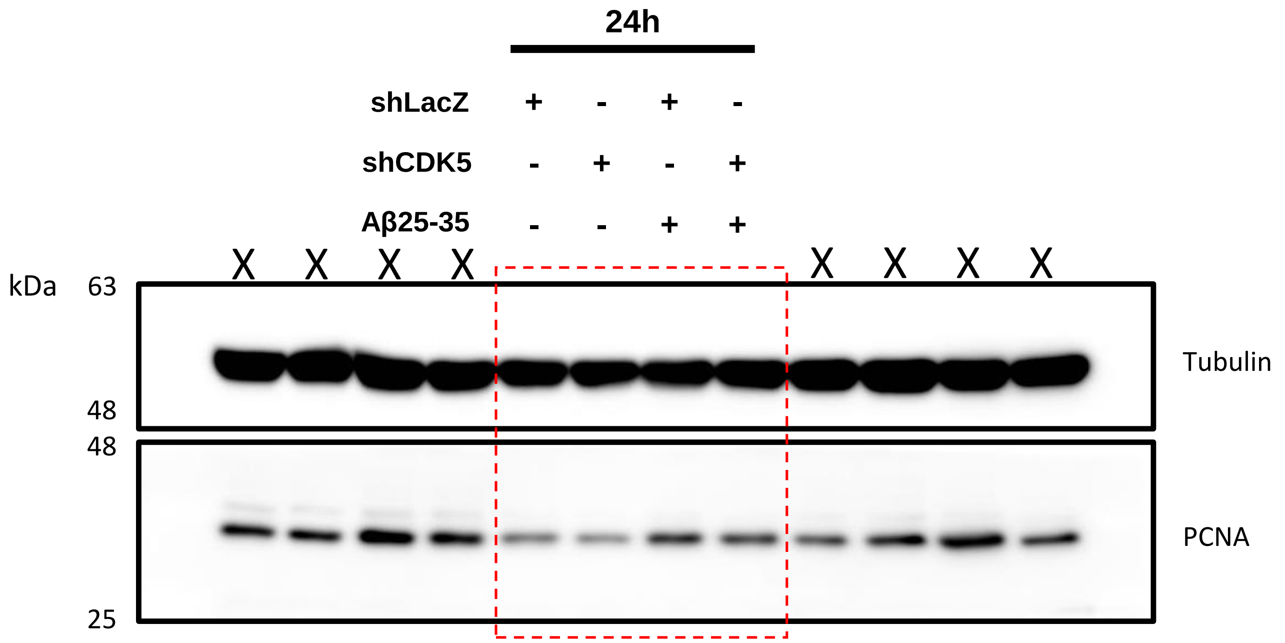


Middle four lanes are shown in the figure; left and right four lanes are for other experiments.

# Figure 4G

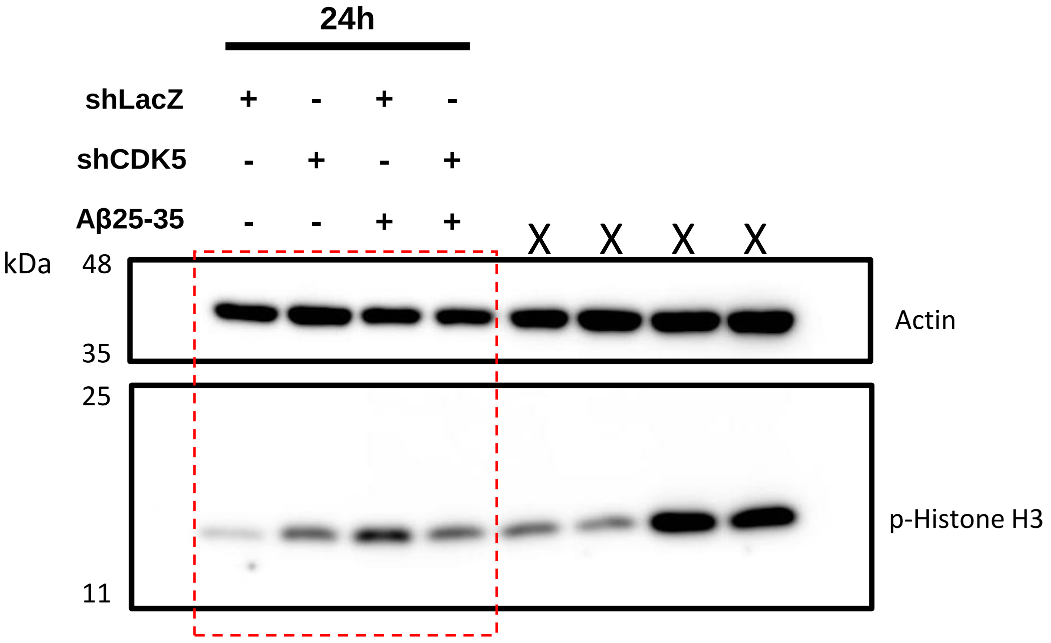


Left four lanes are shown in the figure; right four lanes are for other experiments.

# Figure 4H

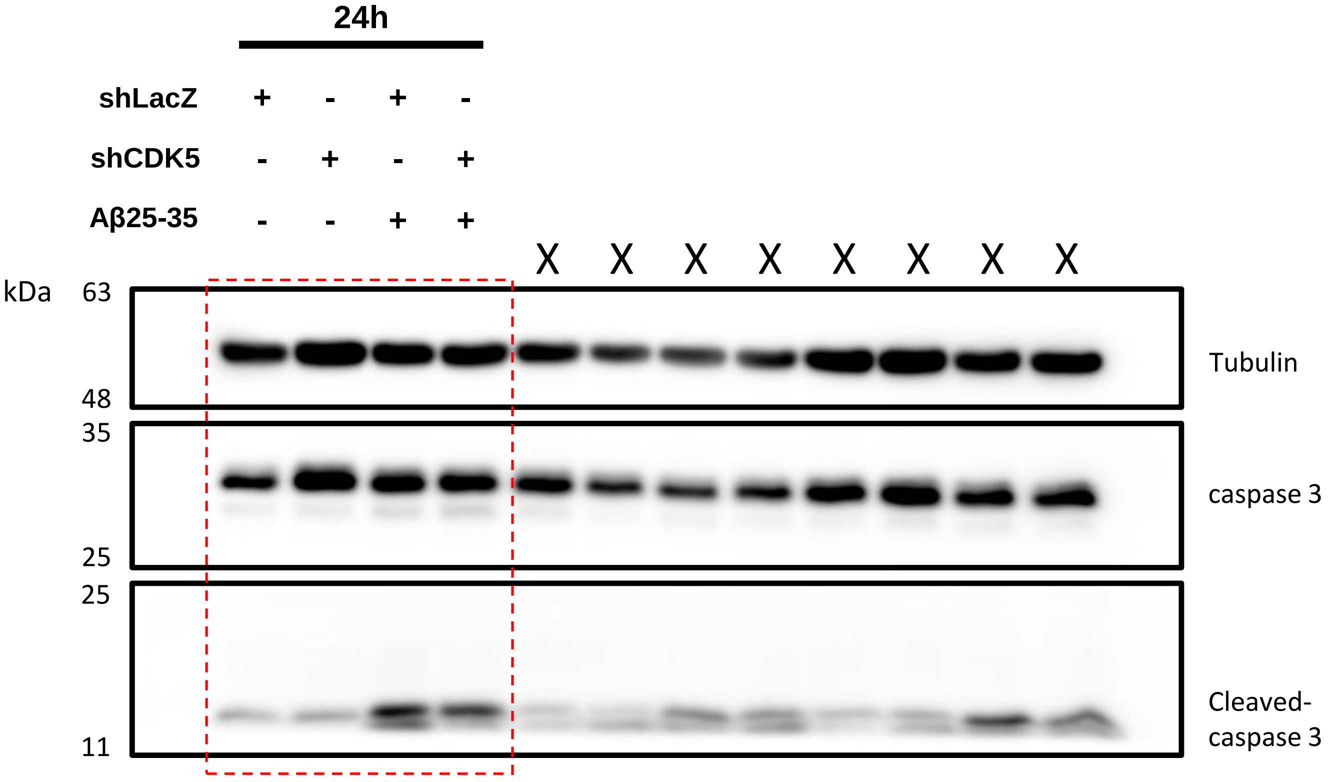


Left four lanes are shown in the figure; right eight lanes are for other experiments.

## Figure 5A

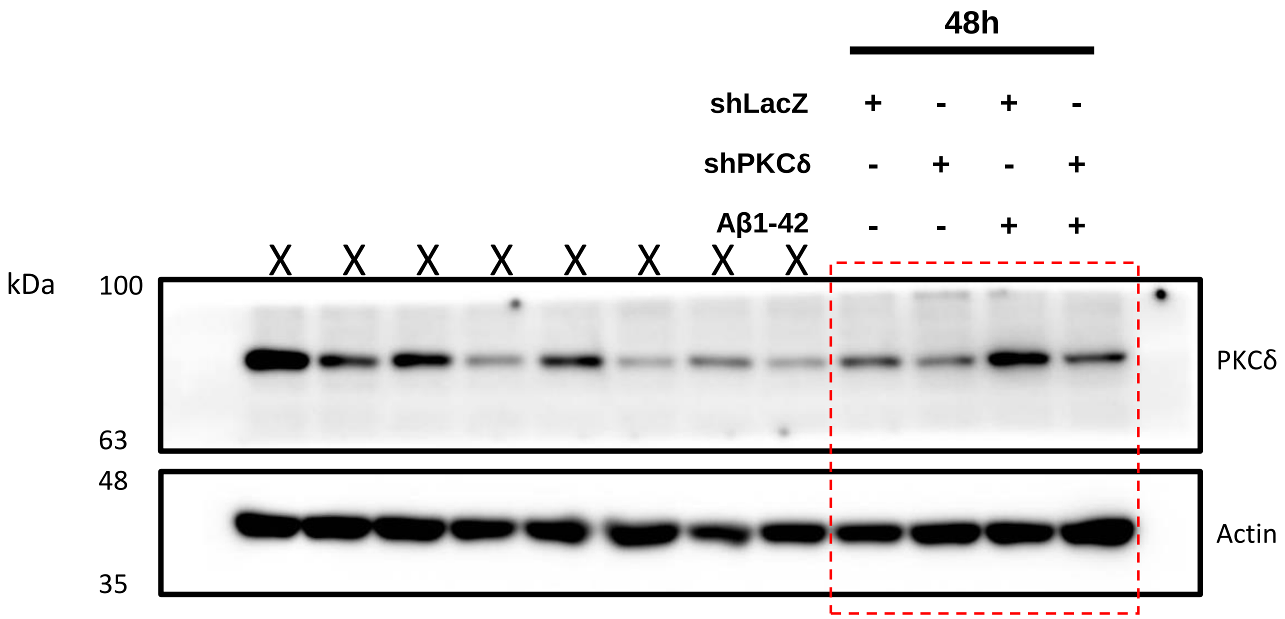


Right four lanes are shown in the figure; left eight lanes are for other experiments.

## Figure 5B

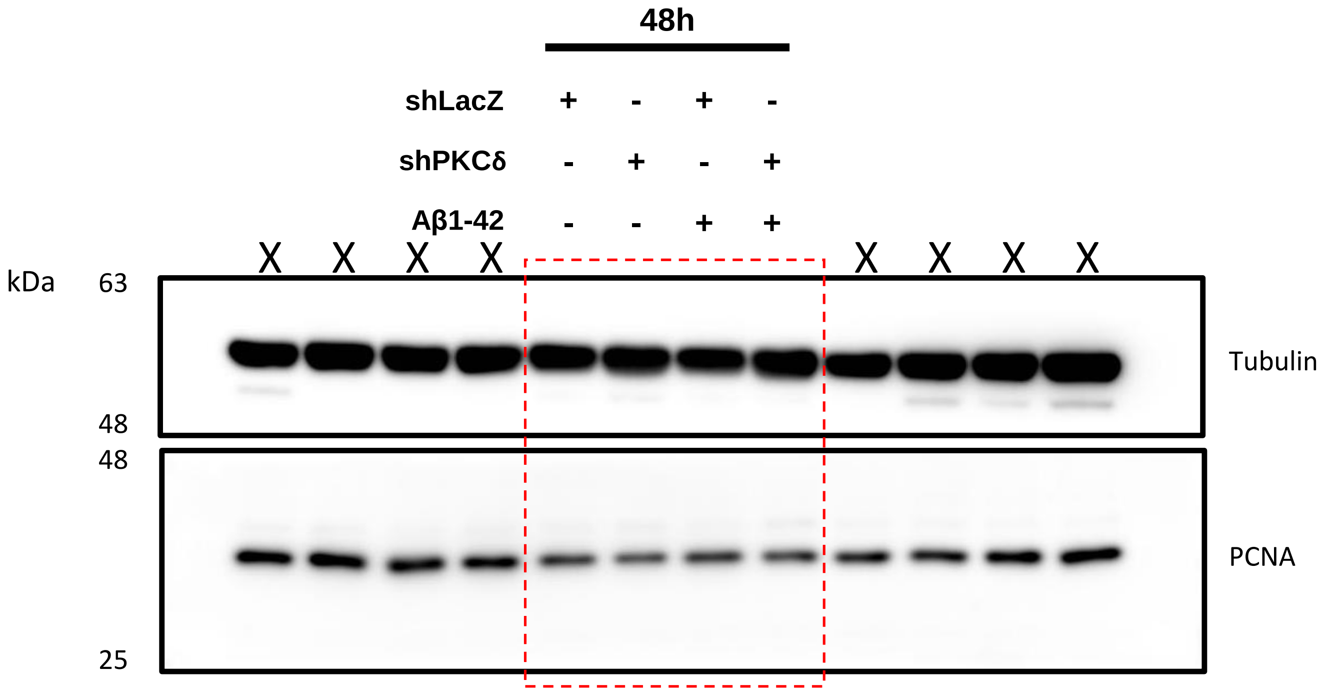


Middle four lanes are shown in the figure; left and right four lanes are for other experiments.

## Figure 5C

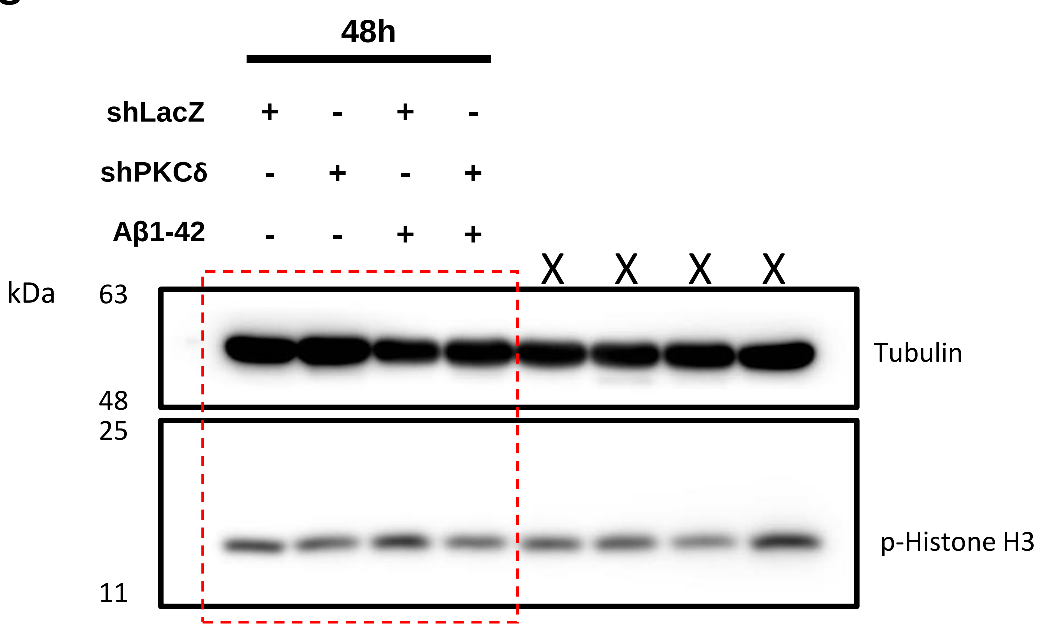


Left four lanes are shown in the figure; right four lanes are for other experiments.

## Figure 5D

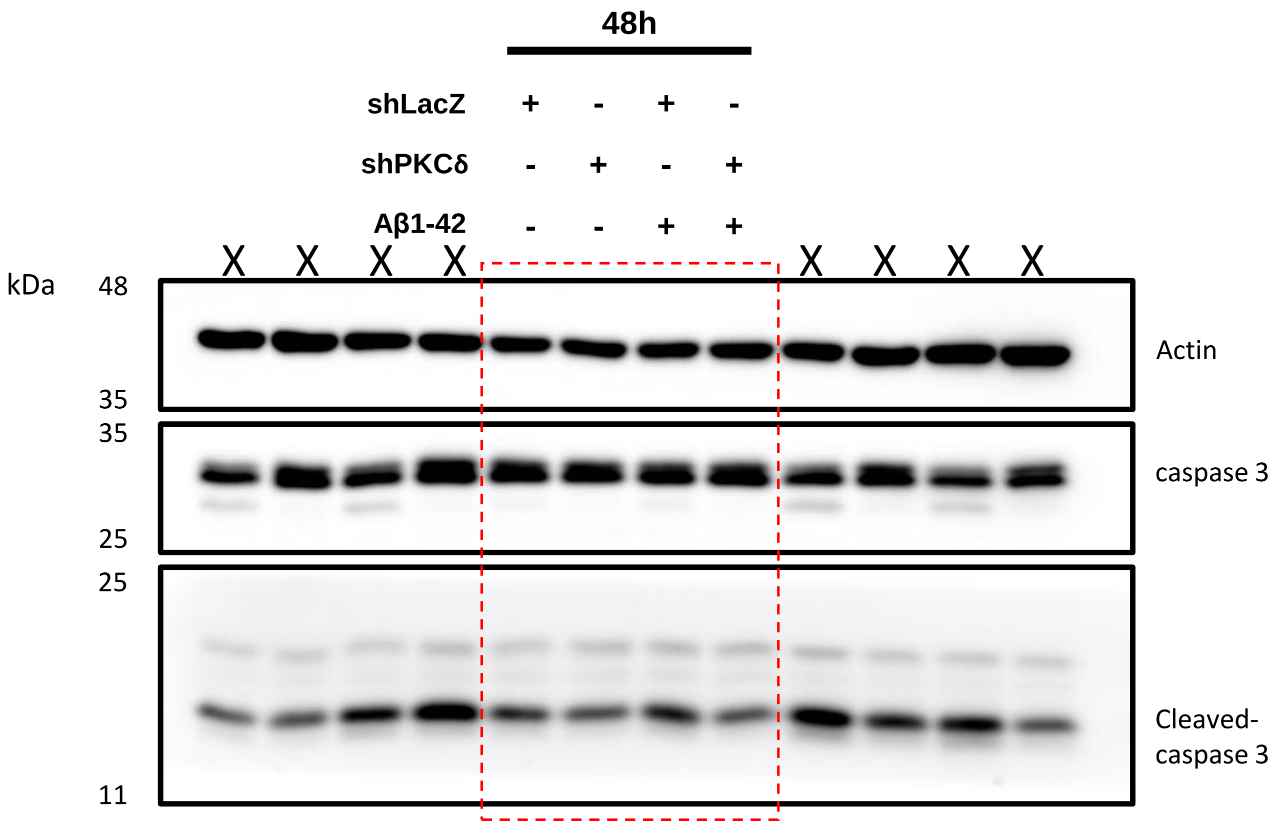


Middle four lanes are shown in the figure; left and right four lanes are for other experiments.

## Figure 6A

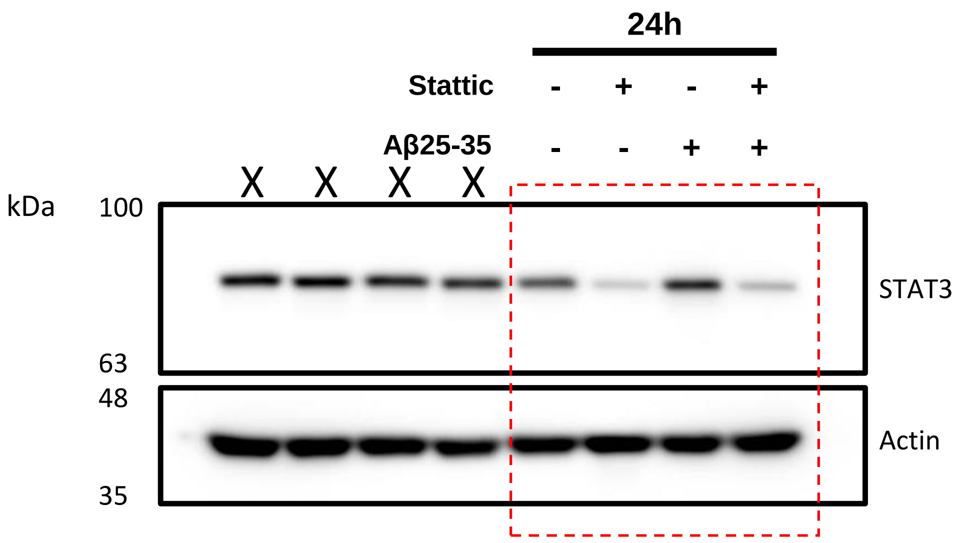


Right four lanes are shown in the figure; left four lanes are for other experiments.

## Figure 6B

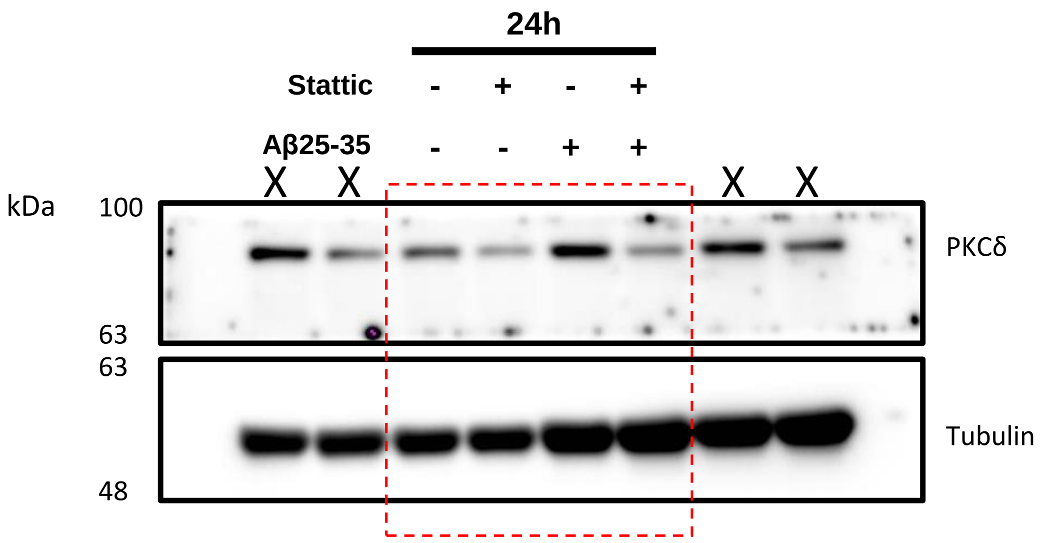


Middle four lanes are shown in the figure; left and right two lanes are for other experiments.

## Figure 6C

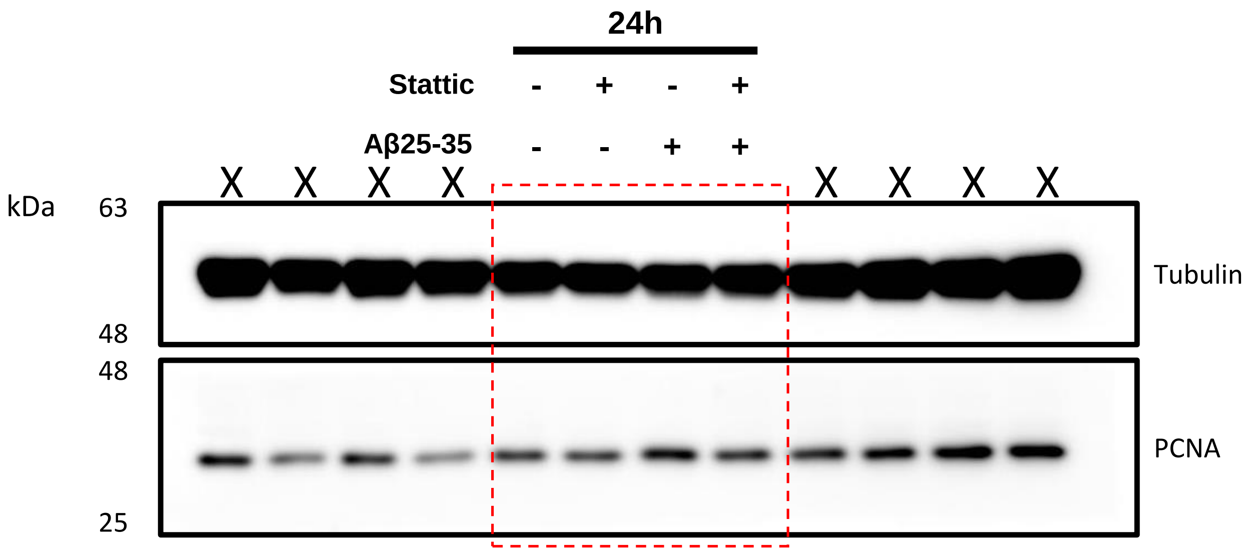


Middle four lanes are shown in the figure; left and right four lanes are for other experiments.

## Figure 6D

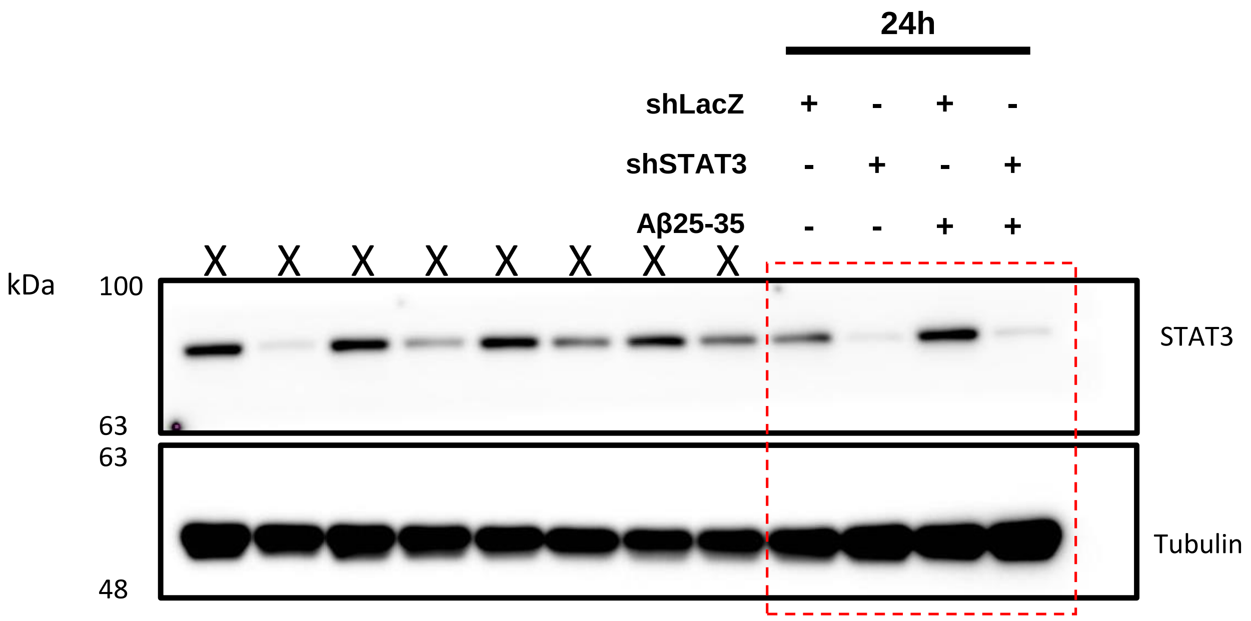


Right four lanes are shown in the figure; left eight lanes are for other experiments.

## Figure 6E

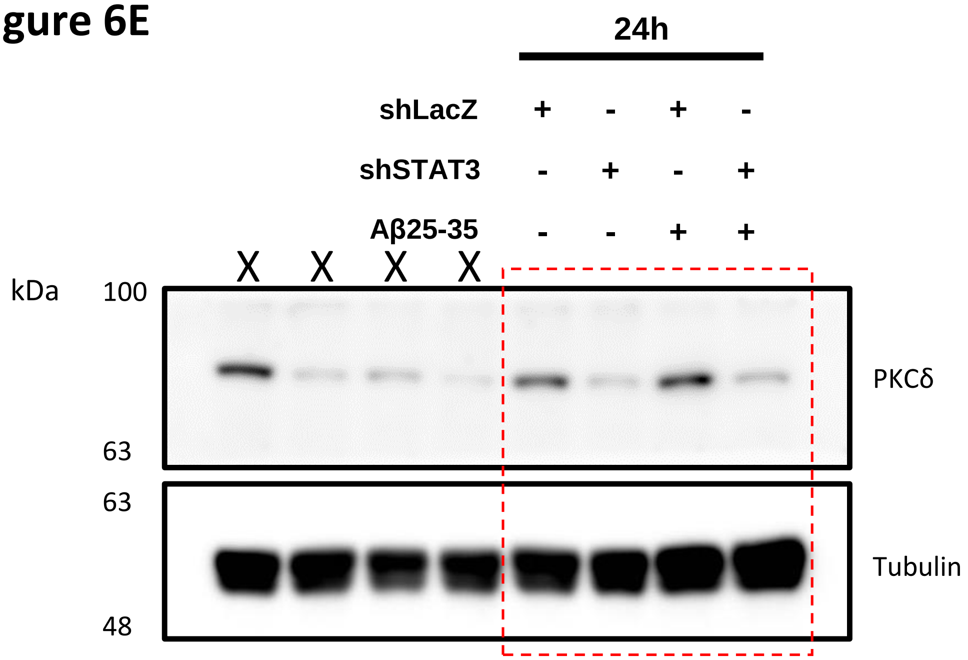


Right four lanes are shown in the figure; left four lanes are for other experiments.

## Figure 6F

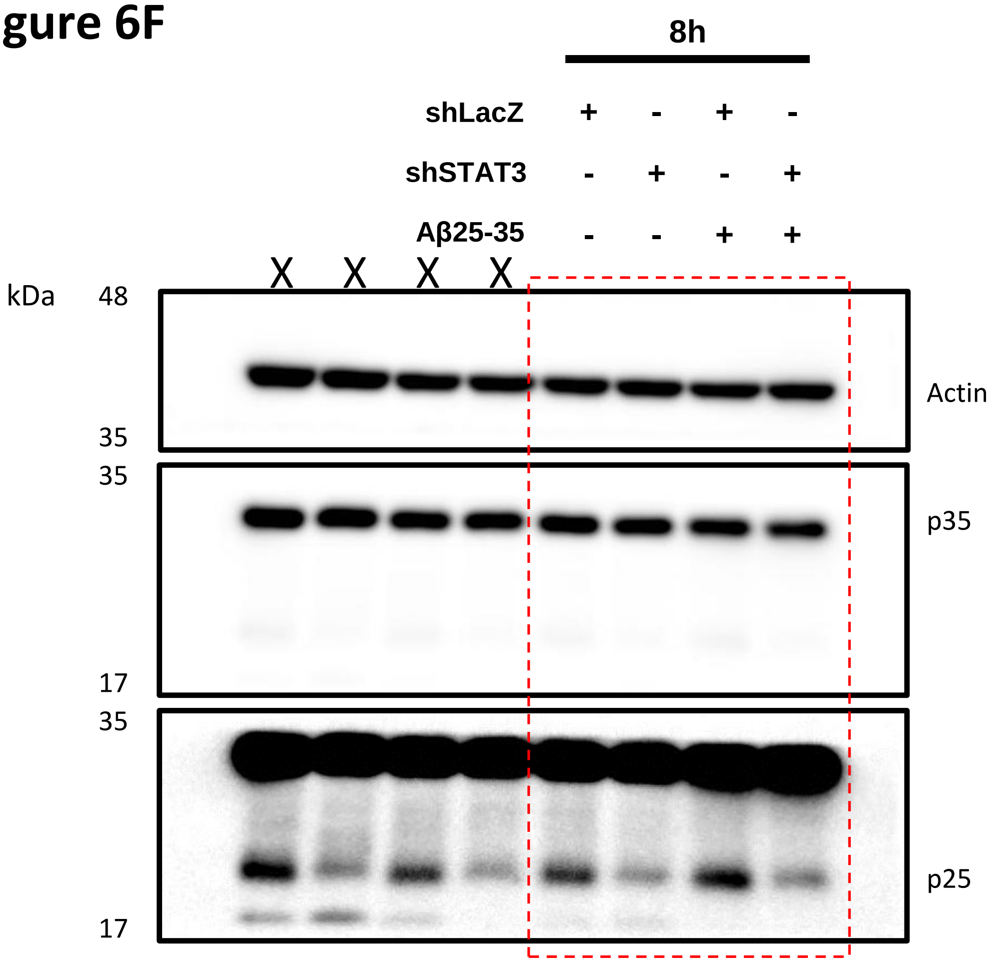


Right four lanes are shown in the figure; left four lanes are for other experiments.

# Figure 6G

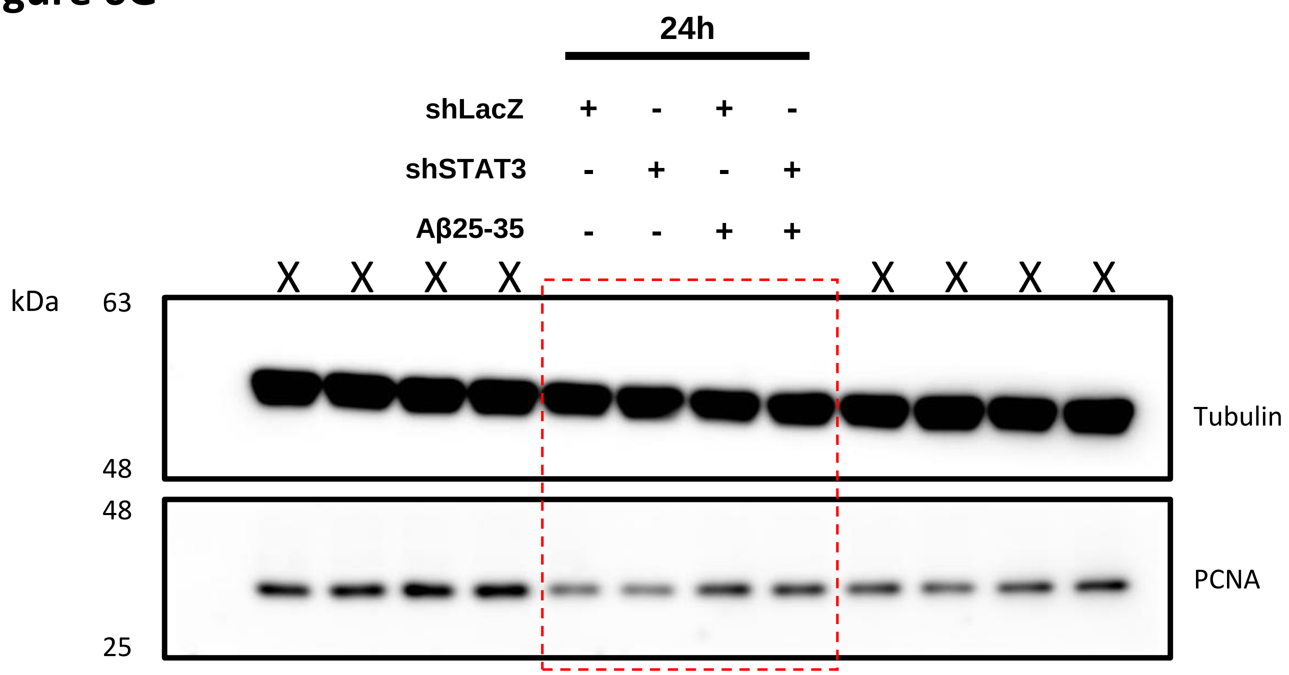


Middle four lanes are shown in the figure; left and right four lanes are for other experiments.

# Figure 6H

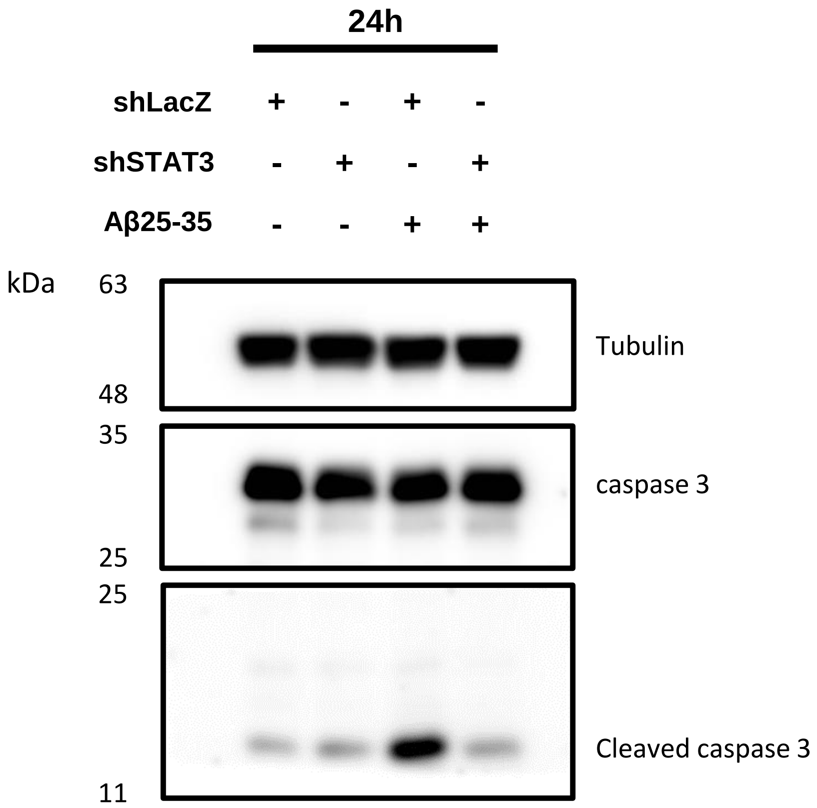

# Figure 7A

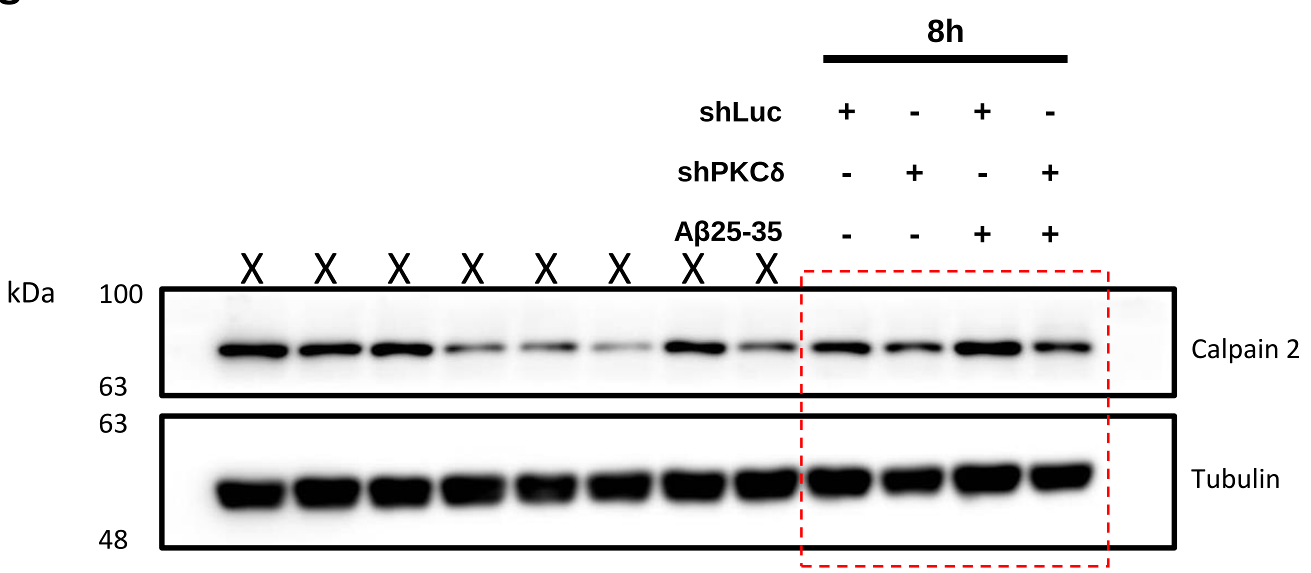


Right four lanes are shown in the figure; left eight lanes are for other experiments.

# Figure 7B

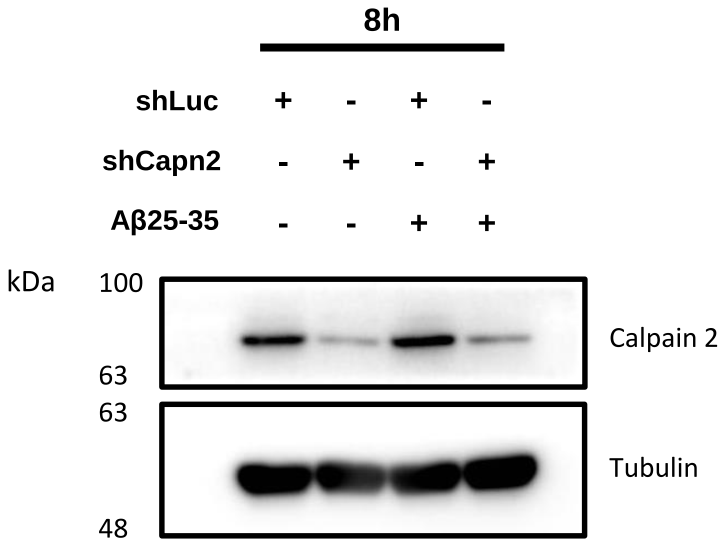

# Figure 7C

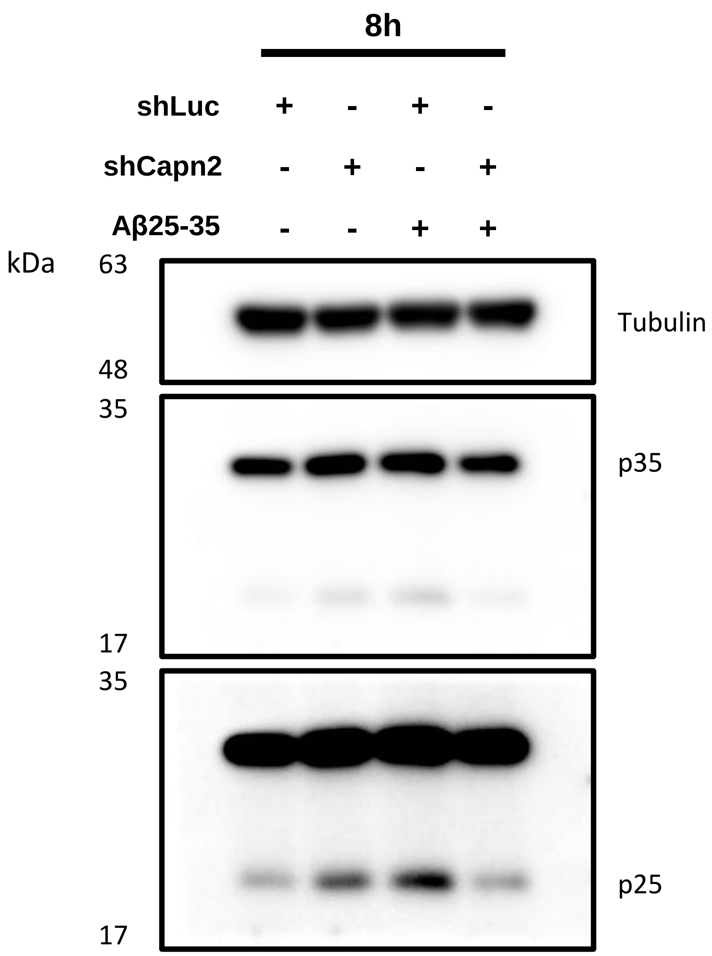


# Figure 7D

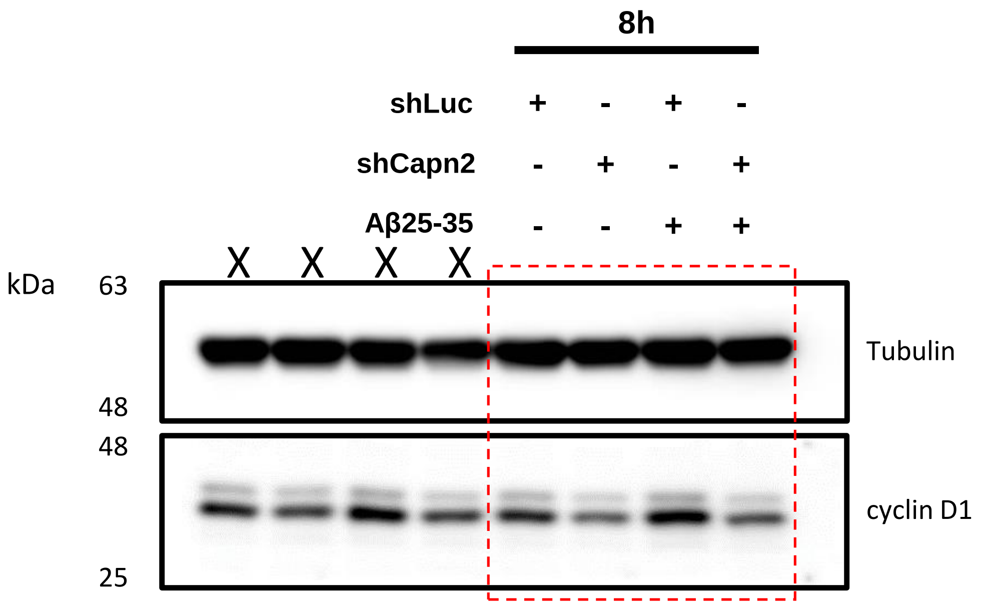


Right four lanes are shown in the figure; left four lanes are for other experiments.

# Figure 7E

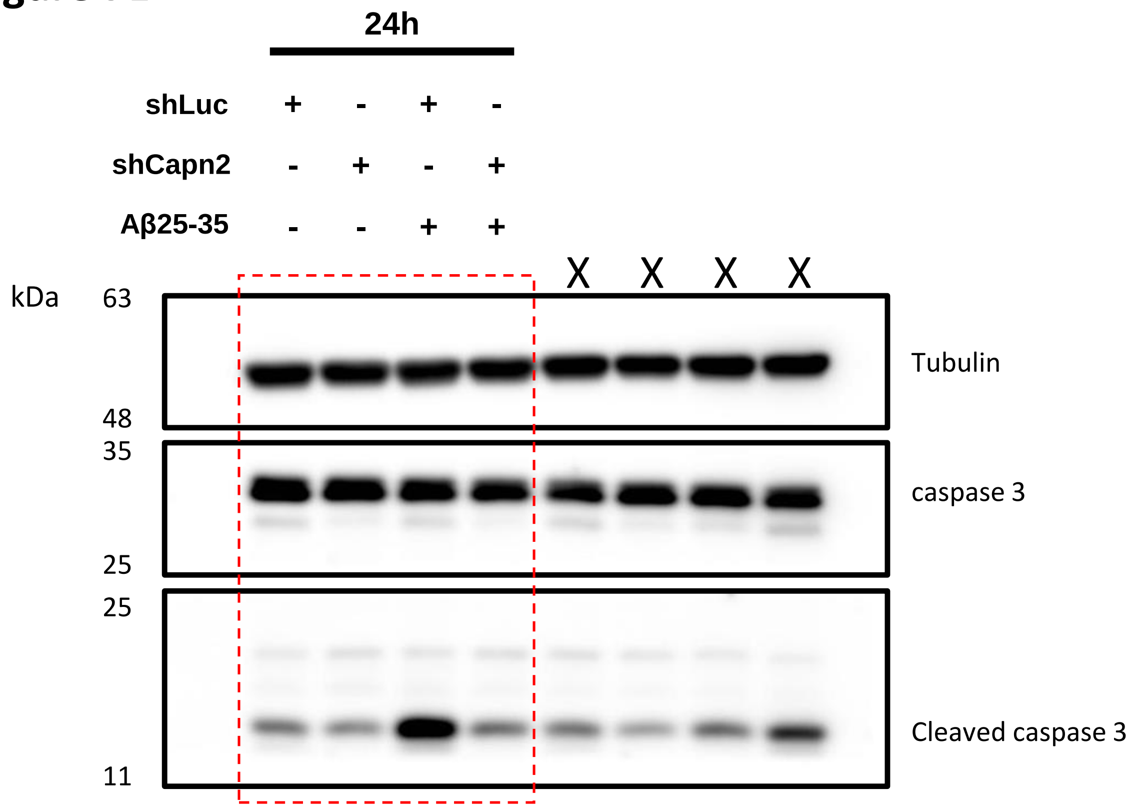


Left four lanes are shown in the figure; right four lanes are for other experiments.

# Figure 7F

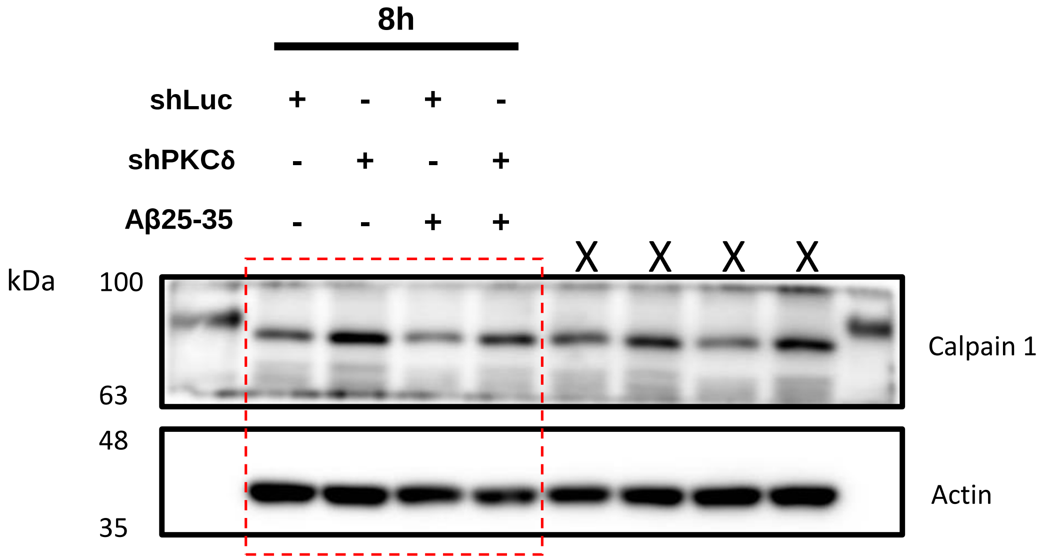


Left four lanes are shown in the figure; right four lanes are for other experiments.

# Figure 7G

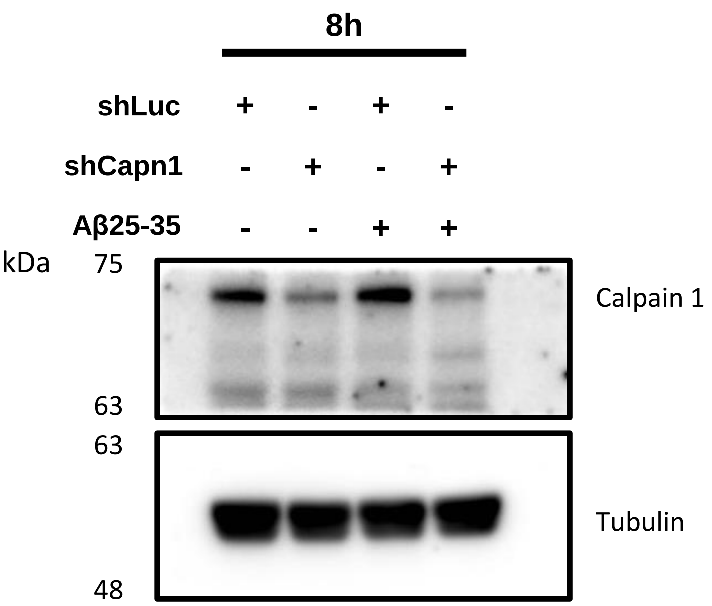


# Figure 7H

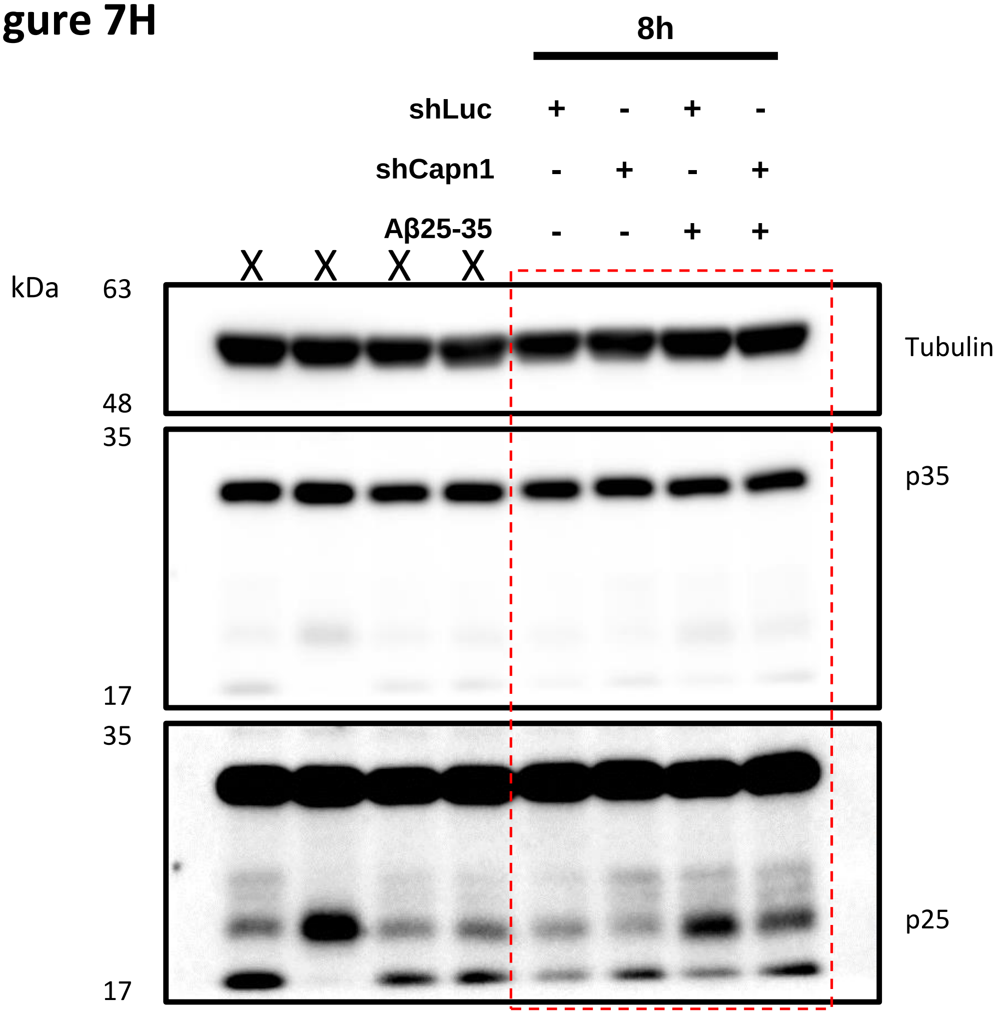


Right four lanes are shown in the figure; left four lanes are for other experiments.

## Figure 7I

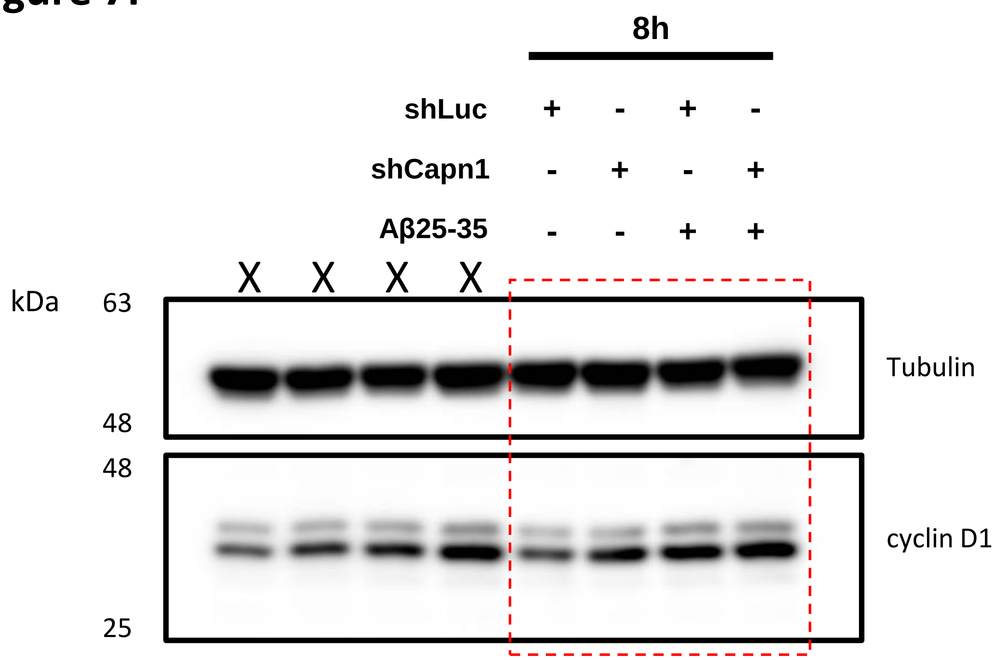


Right four lanes are shown in the figure; left four lanes are for other experiments.

## Figure 7J

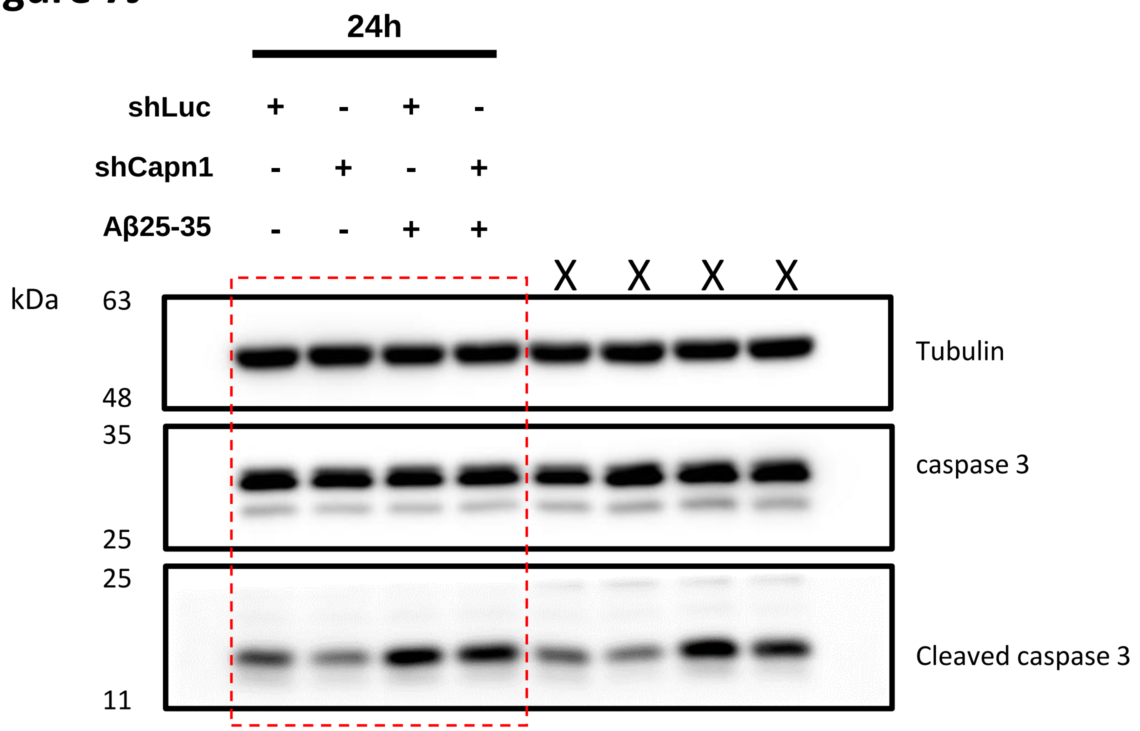


Left four lanes are shown in the figure; right four lanes are for other experiments.

# Figure 8A

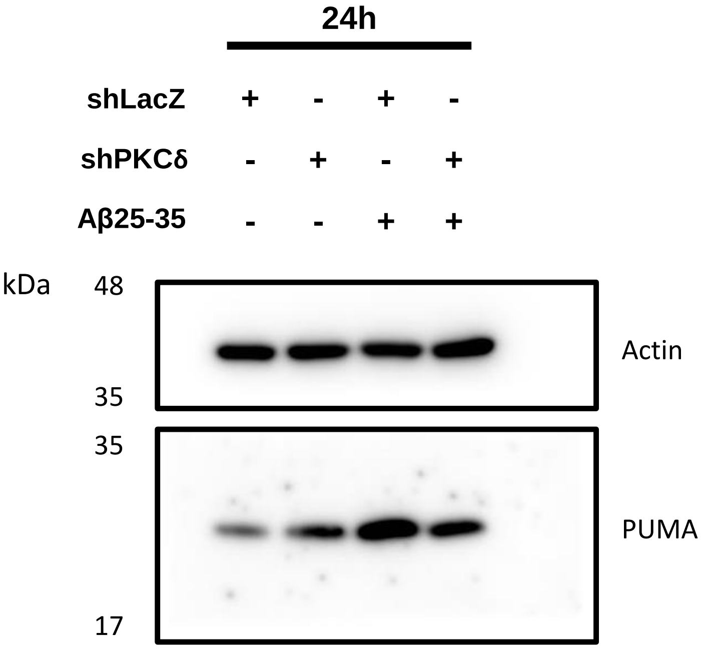


# Figure 8B

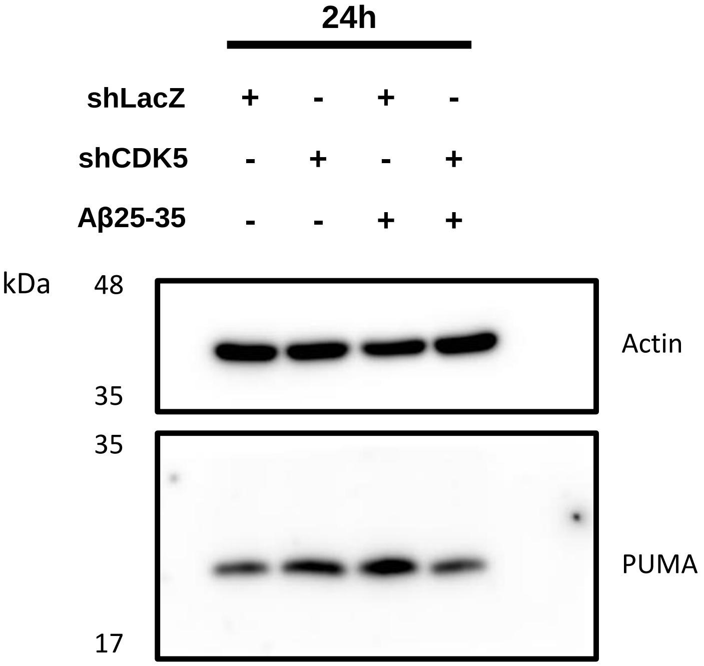

# Figure S1

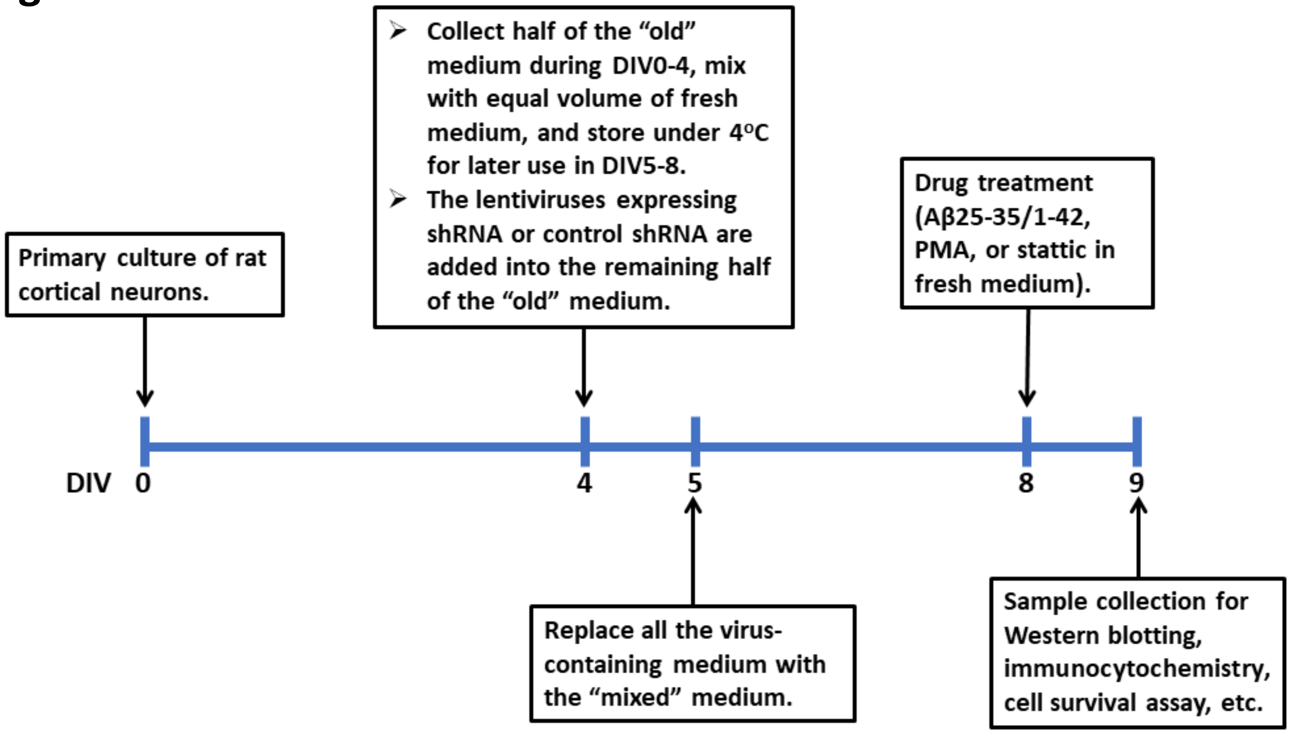


**Figure S1:** A workflow for lentivirus-mediated transfection of shRNA for gene-specific knockdown in primary culture of rat cortical neurons.
